# Synthesis of fully asymmetric diketopyrrolopyrrole derivatives†

**DOI:** 10.1039/d0ra01564d

**Published:** 2021-01-27

**Authors:** Lisa Sharma, Hugo Bronstein

**Affiliations:** Department of Chemistry, University of Cambridge Lensfield Rd Cambridge CB2 1EW USA hab60@cam.ac.uk

## Abstract

Diaryl-diketopyrrolopyrroles (DPP) are a widely studied class of chromophore that possesses unique properties which have been of great interest for use in conjugated polymers and as small molecules in optoelectronic devices. While previously only partially asymmetric DPP derivatives have been reported, here a novel methodology towards fully asymmetric DPP derivatives is demonstrated *via* the synthesis and condensation of novel alkylated thienyl pyrrolinone esters with aromatic nitriles followed by *N*-alkylation. Two fully asymmetric DPP structural isomers T-DPP-P and P-DPP-T were synthesised demonstrating the full customizability of the DPP core. A further two fully asymmetric DPP derivatives incorporating an ethylene glycol chain and a furan moiety were also synthesised, demonstrating the scope of this powerful methodology and it's potential to largely broaden the library of available DPP derivatives.

## Abstract 2

Diaryl-diketopyrrolopyrroles (DPP) are a widely studied class of chromophore that possesses unique properties which have been of great interest for use in conjugated polymers and as small molecules in optoelectronic devices.

## Graphical abstract

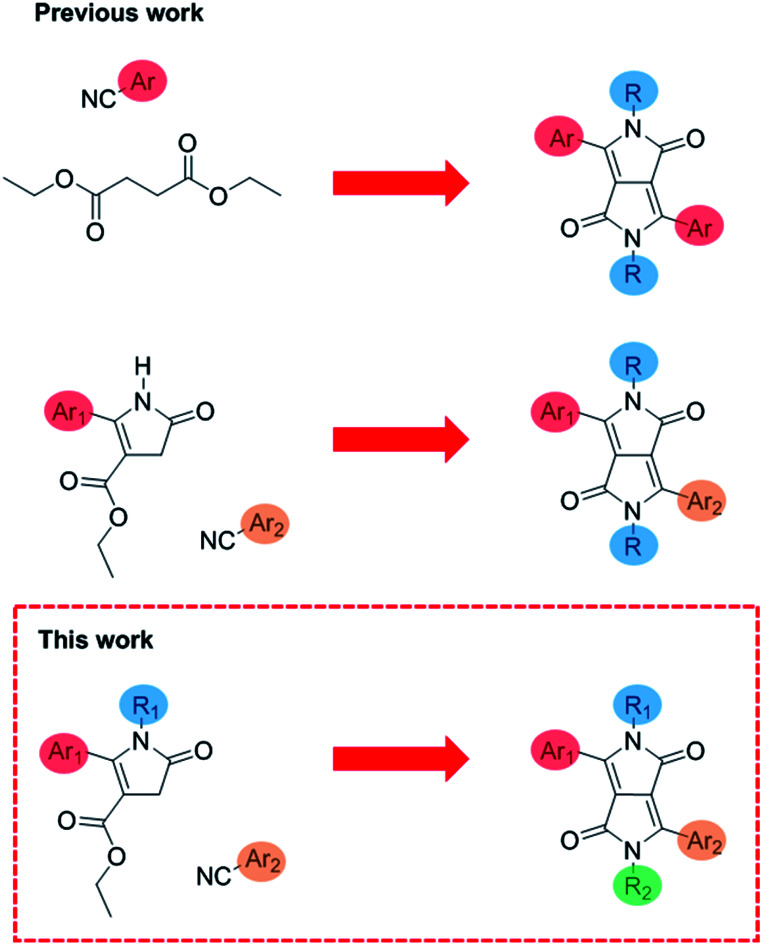

## Introduction

**Fig. 1:**
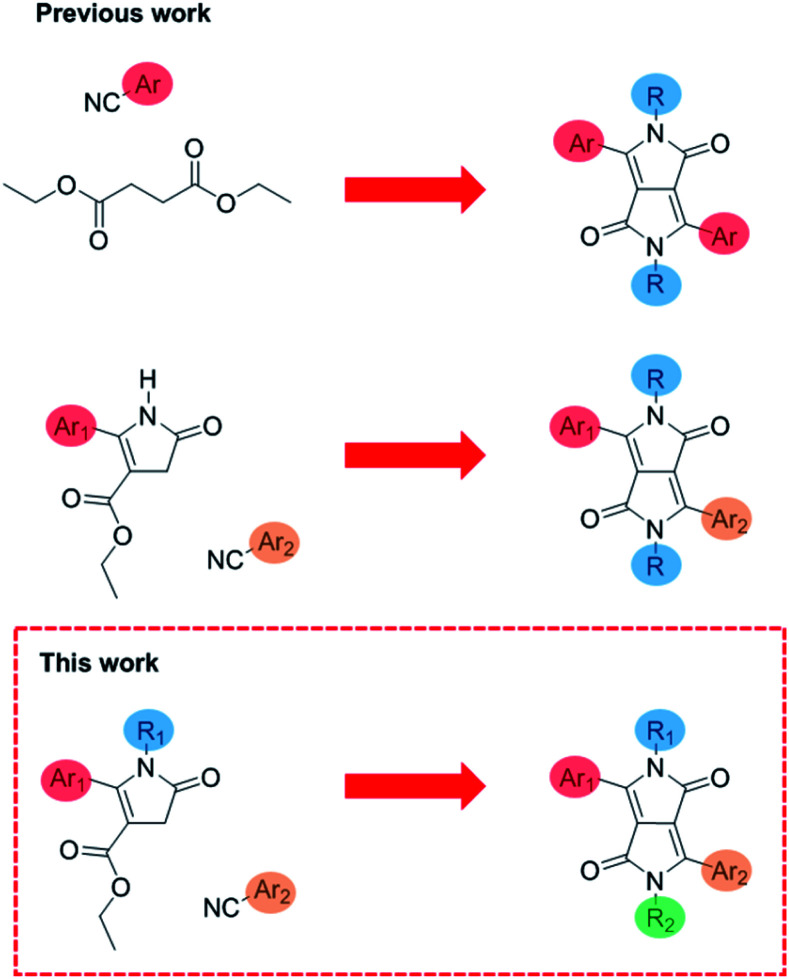
Previous work on symmetrical DPP, translationally asymmetrical DPP^14^ and this work on fully asymmetric DPP.

Diketopyrrolopyrrole is an immensely useful and commonly used organic chromophore used throughout multiple applications. In particular, conjugated polymers and small molecules based on diketopyrrolopyrrole (DPP) have been widely explored and applied in a variety of solution-processed organic electronic devices.^1^ This is due to the electronic deficient nature of the DPP core, allowing the synthesis of narrow band gap donor–acceptor materials.^2^ As DPP is highly planar, this encourages π–π stacking allowing DPP containing conjugated polymers and small molecules to achieve excellent charge-carrier mobilities^3,4^ and high efficiencies in organic solar cells.^5–7^ Its outstanding optical properties (high oscillator strength, tuneable absorption and high photoluminescence quantum yields)^8^ means it has also been used in sensing and bio-imaging applications.^9–11^ DPP consists of a central conjugated bis-lactam core flanked by two aromatic units on either side.^12^ Typically the N atoms of the core are alkylated to provide solubility and control of solid state interactions while the flanking aromatic units are used to fine-tune the optical properties.^13^ DPP is almost exclusively synthesized in a one-pot procedure, which results in a DPP with identical aromatic flanking groups (Fig. 1).^12^ This material is then alkylated with two identical chains, so the resulting unit possesses *C*_2_ symmetry. Recently there has been a growing interest in asymmetric DPP materials, in which the DPP core is flanked by two different aryl groups. There have been various reported examples of asymmetric DPP polymers incorporating a wide variety of a secondary aryl group including thienothiophene,^14^ pyridine,^15^ benzothiophene^16^ and furan^17^ allowing solubility in non-chlorinated solvents while maintaining crystallinity in films, unusual in most common symmetrical DPP polymers. These asymmetric DPP units are made *via* a two-step route through the condensation of thienyl pyrrolinone esters with an aromatic nitrile under basic conditions.^14^

Another interesting class of asymmetric DPP compounds are thioether DPP derivatives (SDPP) reported by Metten,^18^ which are synthesised *via* the cyclization of pyrrolinones with isothiocyanates under basic conditions. These compounds can subsequently undergo cross-coupling with various arylboronic acids *via* the Liebeskind–Srogl reaction.^19^ It is noted however, electron-rich or electron-poor arylboronic acids are reported to give low yields or no reaction product.^18^

As well as translational asymmetry along the conjugated backbone, there have also been examples of dissymmetry of the two solubilising alkyl chains on the DPP unit. Wang *et al.*^20^ have recently reported that by replacing two bulky branched chains (often used to provide suitable solubility for solution processing) on the DPP core for one branched and linear, charge mobility was enhanced *via* reduced steric hindrance, thereby improving planarity of the backbone of the polymer.

Therefore, while various asymmetric DPP units with regard to either different aromatic flanking units or dissimilar alkyl chains, a fully asymmetric DPP unit has not yet been reported due to the prohibitively difficult purifications that would be required. If this were possible it would allow researchers to fine tune both the optical and solid-state properties of DPP units such that their performance across all relevant devices could be improved.

**Fig. 2:**
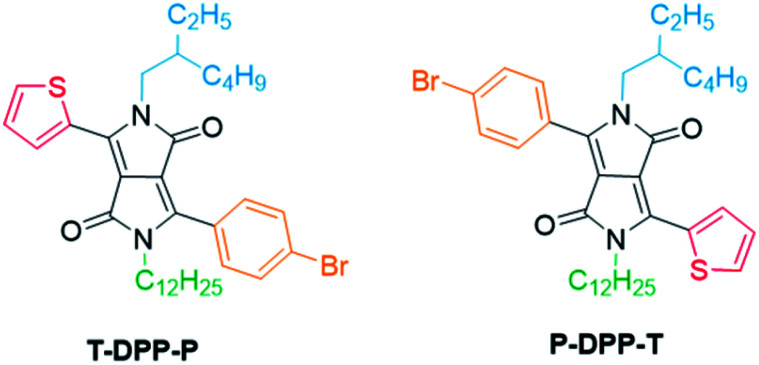
Structures of T-DPP-P and P-DPP-T.

Here we present a novel methodology towards fully asymmetric DPP units (Fig. 2), in which we were able to systematically vary the positioning of the individual solubilising alkyl chains relative to the asymmetric aromatic units. We demonstrate this powerful new method by synthesizing both structural isomers (T-DPP-P and P-DPP-T) of a fully asymmetric DPP and additionally expand the scope to different aromatic groups and alkyl chains.

## Results and discussions

### Synthesis

**Scheme 1:**
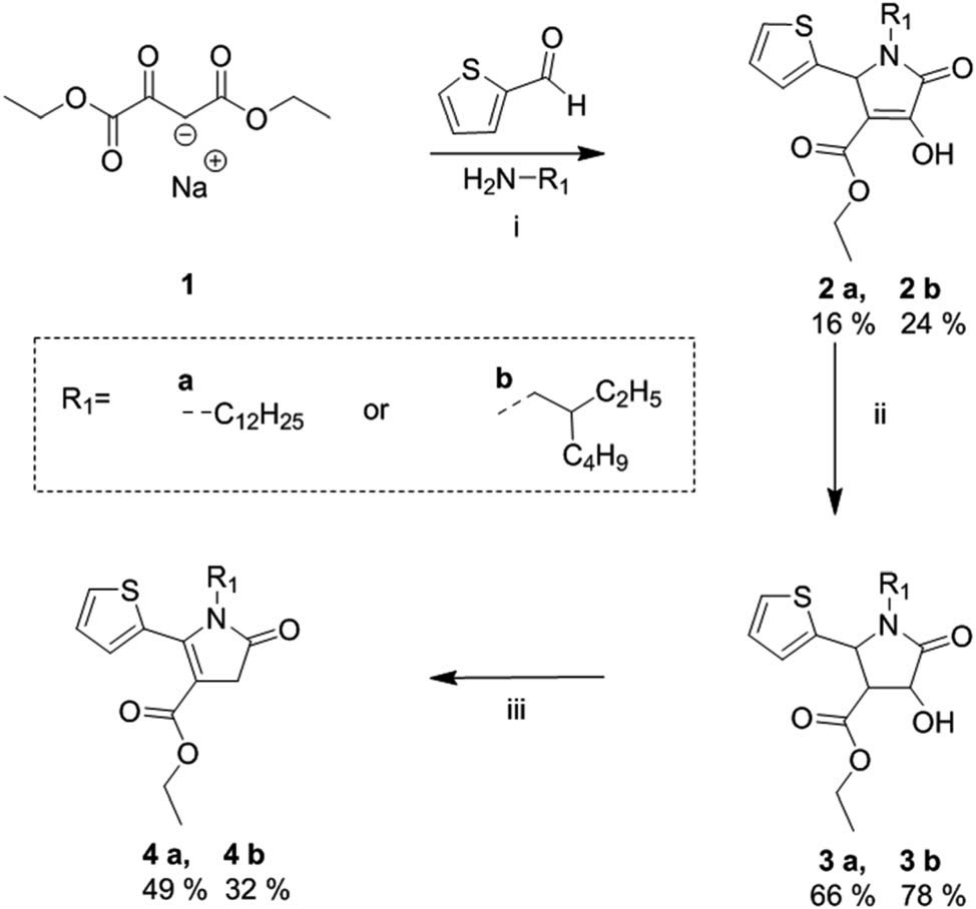
Synthetic route towards alkylated thienyl pyrrolinone esters. (i) EtOH at reflux, 20 h; (ii) Zn powder, a few drops of H_2_SO_4_ in AcOH, at 100 °C, 3 h; (iii) mesyl chloride, TEA, CHCl_3_ at reflux, 30 min.^21^

The synthetic route of the fully asymmetric DPP derivatives, involves the synthesis of novel alkyl-thienyl pyrrolinone esters which are outlined in Scheme 1, based on a previously reported procedure by Metten.^21^ This began with a three-component cyclization of sodium ethyl oxalacetate, 2-thiophenecarboxyaldehyde and alkyl amine to give enols 2a and 2b. This was followed by a zinc reduction to give alcohols 3a and 3b, which were subsequently mesylated and treated with TEA to the give the desired alkyl-thienyl pyrrolinone esters 4a and 4b in reasonable yield, which were used as building blocks towards the synthesis of the asymmetric DPP units.

**Scheme 2:**
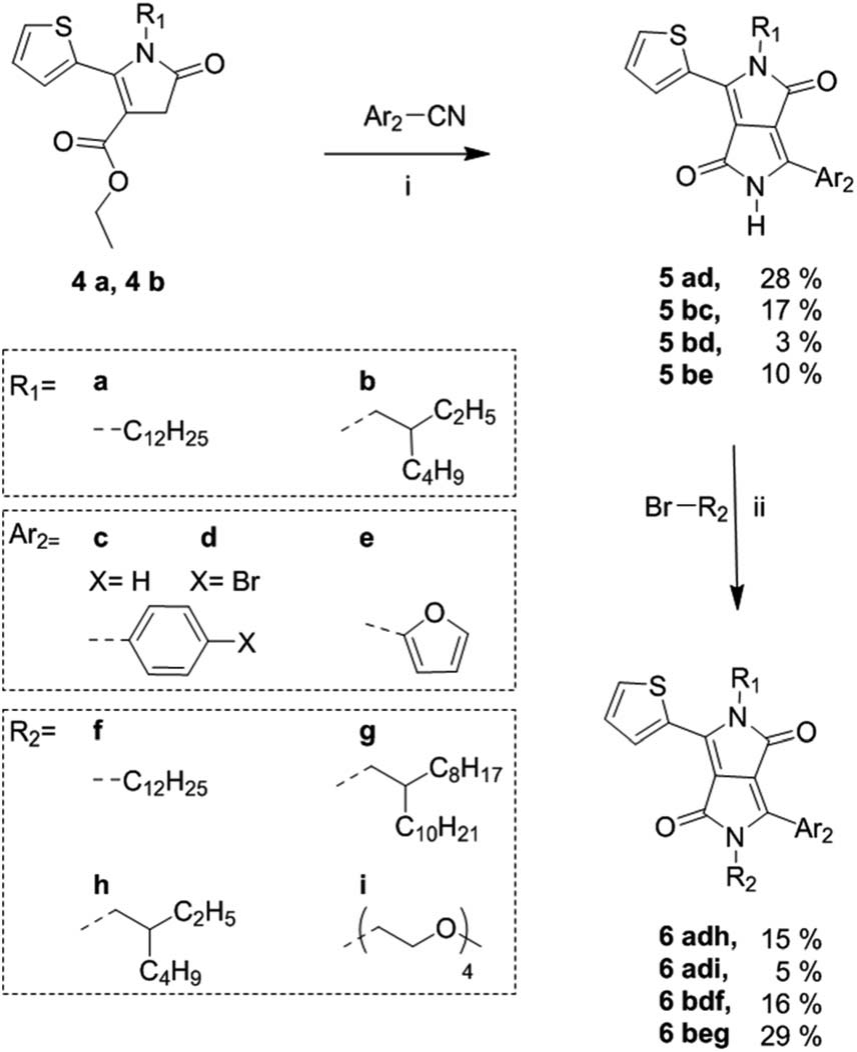
Synthetic route to fully asymmetric DPP cores from alkylated thienyl pyrrolinone esters. (i) Na/FeCl_3_, 2-methyl-2-butanol at reflux, 2–18 h; (ii) K_2_CO_3_, 18-crown-6, DMF at 120 °C, 18 h.

Following the synthesis of the alkyl-pyrrolinone ester building blocks, was condensation with either benzonitrile, 4-bromobenzonitrile or 2-furanitrile to give the asymmetric DPP cores as shown in Scheme 2.

The brominated species 5ad and 5bd were also synthesised to demonstrate the potential of these materials as precursors for subsequent polymerisations. Optimized reaction conditions for the synthesis of 5ad were investigated and are summarised in Table S1 in the ESI.† The optimized conditions were then applied to the synthesis of 5bc and 5bd. Derivative 5be was synthesised based on previously reported conditions for the synthesis of an unsubstituted thieno[3,2-*b*]thiophene/furan asymmetric DPP derivative.^17^ However, it was observed that the condensation step gave a poor yield particularly when reacting with the branched thienyl pyrrolinone ester 4b with 4-bromobenzonitrile. It was observed that in the presence of an excess amount of NaO*t*-Am, 4a and 4b showed some signs of degradation. It is possible that through future investigation of alternative weaker bases such as LiO*t*Bu, degradation of the pyrrolinone esters may be prevented and the yield can be improved. Derivatives 5ad, 5bc and 5bd were purified *via* washing with MeOH. While 5ad and 5bc were obtained in high purity, some impurities remained in 5bd. Derivative 5be underwent partial purification *via* column chromatography, however, due to insufficient solubility and a high polarity (as a result of the short alkyl chain and the unsubstituted amide group) this led to large product loss on silica. Thus, 5bd and 5be were used in the next step with no further purification. In general, as previously reported for the synthesis of symmetric and asymmetric DPP cores, purification *via* column chromatography does not occur until subsequent di-alkylation, to ensure sufficient solubility.^2,14^ Finally, the asymmetric DPP units were obtained in modest yields following *N*-alkylation with either an alkyl or ethylene glycol bromide chain. The low yields following alkylation were in part due to obtaining the undesired *N*,*O*-alkylated side product as well as the *N*,*N*-alkylated product. In general, it is often reported that the alkylation of DPP compounds are of low yield.^12,22,23^

To demonstrate the scope of this new methodology as well as the structural isomers (T-DPP-P and P-DPP-T), we also synthesised two additional asymmetric DPP compounds, 6adi which incorporates both an alkyl and oligo ethylene glycol chain allowing potential use in sensing applications which have been reported for such compounds,^24^ as well as 6beg which incorporates a furan moiety, further improving solubility. It was however observed, 6beg showed poor stability in which it degraded rapidly upon contact on silica during purification and exposure to light in solution.

### Optical properties

**Fig. 3:**
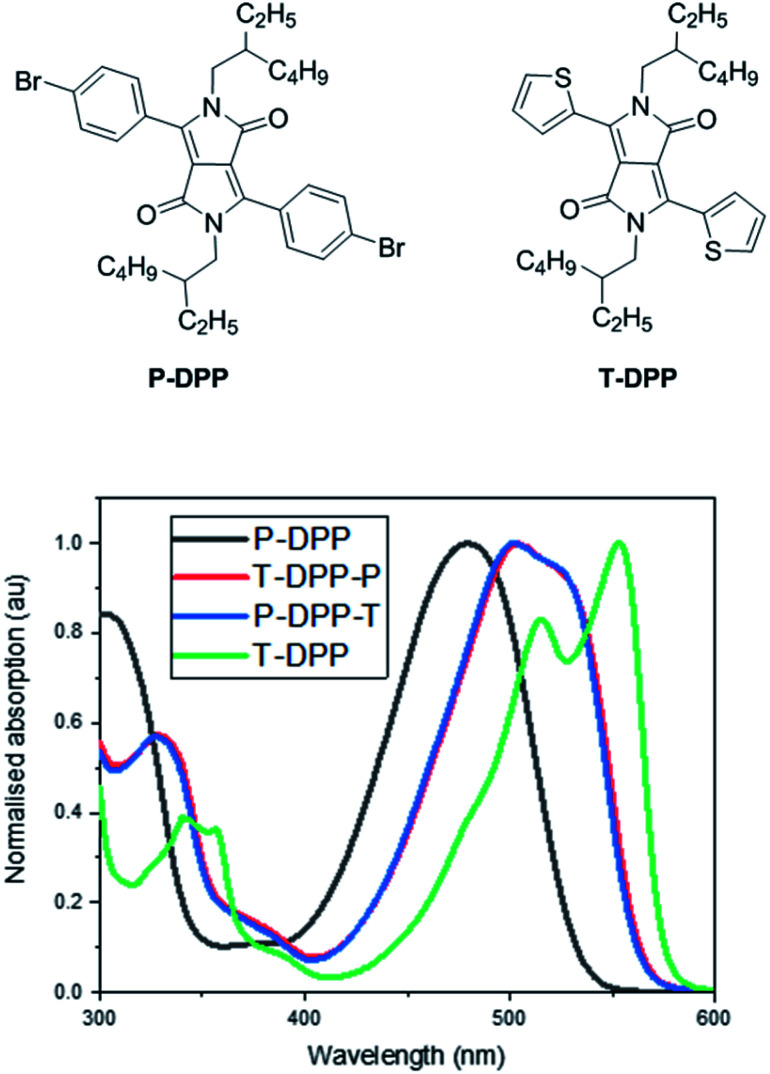
Normalized solution (chlorobenzene ∼ 4 μg mL^−1^) absorption spectra for P-DPP, T-DPP, T-DPP-P and P-DPP-T. Path length 10 mm. The molecular molar extinction coefficients (*ε*) were 110 637 cm^−1^ L mol^−1^, 115 389 cm^−1^ L mol^−1^ and 90 525 cm^−1^ L mol^−1^ for P-DPP, T-DPP/T-DPP-P and T-DPP respectively.

The solution UV-vis absorption spectra of T-DPP-P and P-DPP-T in chlorobenzene (∼4 μg mL^−1^) are shown in Fig. 3. The absorption spectra of P-DPP and T-DPP are also shown below for reference and were synthesised based on previously reported literature.^2,25,26^ The absorption spectra for P-DPP-T and T-DPP-P are observed to be almost identical as a result of the compounds being structural isomers. In comparison, the P-DPP absorption is blue shifted and broad likely as a result of the greater torsional angle between the DPP core and phenyl moieties, as observed in the calculated structure. On the other hand, T-DPP absorption is the most red shifted and sharpest of the structures, likely due to the enhanced conjugation as a result of the smaller torsional angle between the thiophene units and DPP core as seen in the calculated structure. Isomers T-DPP-P and P-DPP-T absorptions lie between that of P-DPP and T-DPP, as the compounds contain one thiophene and one phenyl moiety each.

The HOMO and LUMO levels calculated for the structural isomers T-DPP-P/P-DPP-T are −5.32 eV and −2.78 eV. In comparison P-DPP and T-DPP calculated HOMO and LUMO levels are observed at −5.51 eV, −2.86 eV and −5.13 eV, −2.68 respectively. Again, the HOMO and LUMO levels of the asymmetric isomers are observed to lie between that of the two symmetrical DPP reference compounds, demonstrating the ability to fine tune the absorption and bandgap *via* synthesis of asymmetric DPP derivatives. Calculated structures for P-DPP, T-DPP, and T-DPP-P/P-DPP-T as well as HOMO and LUMO levels are included in the ESI.† It is to be noted that the energy minimized structures have been obtained using a structurally modified model where both the alkyl chains have been replaced with methyl groups for simplicity.

## Conclusion

In conclusion, we have designed a novel methodology towards synthesising fully asymmetric DPP derivatives demonstrated through the synthesis of four novel fully asymmetric DPP compounds. We have demonstrated it is possible to systematically synthesise structural isomers of asymmetric DPP units, allowing future investigation on the effect of alkyl–aryl positioning on the properties and performance of DPP based conjugated polymers and small molecules, in optoelectronic devices. We have also shown that the scope of this methodology extends to further aromatic groups such as furan and the possibility to introduce potential sensing applications to these compounds *via* incorporation of an oligo ethylene glycol chain, as reported for conjugated polymers incorporating similar structures.^24^ This new method also allows the possibility to synthesise previously unavailable DPP derivatives, allowing the possibility to greatly broaden the library of existing DPP compounds, thus opening new horizons towards novel and unique DPP structures and their application in optoelectronics.

## Experimental section

### General experimental

All moisture and air sensitive reactions were carried out in oven dried flasks under an inert argon atmosphere. RT refers to 25 °C maintained by the use of a heating mantle. All reactions were covered with foil unless otherwise stated and were magnetically stirred. Merck Geduran® Si 60 silica gel or Biotage® Isolera™ four with either Biotage® SNAP/SNAP Ultra cartridges (10 g, 20 g, 50 g or 100 g) were used for column chromatography during purification. DC Fertigfolien ALUGRAM aluminium sheets coated with silica gel, were used for carrying out analytical thin layer chromatography (TLC). Compounds were visualised by ultra-violet light. Chemicals were used as supplied. Anhydrous solvents were supplied commercially and used under an inert argon atmosphere. All other solvents and reagents used were supplied commercially and used as received.

### Instrumental techniques

^1^H NMR were carried out at 400 MHz on an Avance III 400 HD Spectrometer or at 600 MHz on an Avance 600 BBI Spectrometer at the Department of Chemistry, University of Cambridge. The internal standard used was CHCl_3_ (*δ* = 7.26 ppm, s) and DMSO (*δ* = 2.50 ppm, s). ^1^H NMR shifts were reported to the nearest 0.01 ppm and the following abbreviations were used: s, singlet; d, doublet; t, triplet; q, quartet; qn, quintet; sxt, sextet; m, multiplet; br, broad; Ar, aromatic; Th, thienyl; Ph, phenyl. The coupling constants (*J*) are measured in Hertz. ^13^C NMR spectra were recorded at 125 MHz on a BRUKER DCH Cryoprobe Spectrometer in the stated solvent. The internal standard used was ^13^C NMR (*δ* = 77.2 ppm, t) and (CD_3_)_2_SO (*δ* = 39.52 ppm, s). ^13^C NMR chemical shifts are reported to the nearest 0.1 ppm. Mass spectra were obtained using a Waters LCT, Finnigan MAT 900XP or Waters MALDI micro MX spectrometer at the Department of Chemistry, University of Cambridge. UV-vis spectra were recorded on a Shimadzu UV-1800 spectrophotometer using Hellma® absorption cuvettes in chlorobenzene (∼4 μg mL^−1^), 200–2500 nm spectral range, pathlength 10 mm, chamber volume 3500 μL. The molecular molar extinction coefficient (*ε*) was calculated according to the Beer Lambert law *A* = *ε*·*l*·*c*.

### Ethyl-1-dodecyl-4-hydroxy-5-oxo-2-(thiophen-2-yl)-2,5-dihydro-1*H*-pyrrole-3-carboxylate 2a

To an oven dried flask under argon equipped with a reflux condenser was added sodium alkyl oxalacetate (50 g, 237.90 mmol), dodecylamine (44 g, 237.90 mmol), ethanol (313 mL) and 2-thiophenecarboxaldehyde (26.68 g, 237.90 mmol). The reaction mixture was then heated under reflux and allowed to stir for 20 h. The reaction was allowed to cool to RT followed by the addition of water (400 mL) and acidified to pH 2 *via* dropwise addition of H_2_SO_4_. The white solid that precipitated out of solution was collected *via* filtration and was washed with hexane to afford the product as a white solid (16 g, 38.0 mmol, 16%). ^1^H NMR (400 MHz, DMSO) *δ* 7.49 (d, *J* = 5.0 Hz, 1H, ThH), 7.24 (d, *J* = 3.3 Hz, 1H, ThH), 7.00 (dd, *J* = 5.0, 3.3 Hz, 1H, ThH), 5.57 (s, 1H, C*H*NC_12_H_25_), 4.14–3.92 (m, 2H, OC*H*_2_CH_3_), 3.52–3.43 (m, 1H, alkyl chain), 2.75 (ddd, *J* = 13.5, 8.0, 5.3 Hz, 1H, alkyl chain), 1.53–1.10 (m, 20H, alkyl chain), 1.07 (t, *J* = 7.1 Hz, 3H, OCH_2_C*H*_3_), 0.86 (t, *J* = 6.7 Hz, 3H, alkyl chain). ^13^C NMR (101 MHz, DMSO) *δ* 164.5, 162.4, 154.9, 141.1, 128.6, 127.3, 126.5, 110.9, 59.8, 56.1, 31.8, 29.5, 29.4, 29.3, 29.2, 29.0 27.7, 26.6, 22.6, 14.5, 14.4. HRMS (TOF MS ASAP+): calculated for C_23_H_36_NO_4_S^+^: 422.2365. Found *m*/*z* 422.2362 [M + H]^+^.

### Ethyl-1-(2-ethylhexyl)-4-hydroxy-5-oxo-2-(thiophen-2-yl)-2,5-dihydro-1*H*-pyrrole-3-carboxylate 2b

Compound 2b was made using the same procedure as making 2a with the following reagents: sodium alkyl oxalacetate (40.00 g, 190 mmol), 2-ethyl-1-hexylamine (24.60 g, 190 mmol), ethanol (250 mL) and 2-thiophenecarboxaldehyde (21.35 g, 190 mmol). The organic layer was then extracted with chloroform × 3 and dried over MgSO_4_. The resulting solution was filtered and concentrated *in vacuo* to afford the dark brown oil crude product, which was partially purified *via* column chromatography on silica gel (hexane : chloroform = 50 : 50), (chloroform 100%) and finally (chloroform : ethyl acetate = 50 : 50) to give an off white solid which was collected *via* filtration. This was then washed with hexane to afford the pure product as a white solid (16.65 g, 44.5 mmol, 24%). ^1^H NMR (400 MHz, DMSO) *δ* 11.71 (br s, 1H, OH), 7.51 (d, *J* = 5.0 Hz, 1H, ThH), 7.24–7.21 (m, 1H, ThH), 7.04–6.99 (m, 1H, ThH), 5.55 (d, *J* = 5.2 Hz, 1H, C*H*NC_8_H_17_), 4.15–3.95 (m, 2H, OC*H*_2_CH_3_), 3.46–3.34 (m, 2H, NC*H*_2_), 1.51 (s, 1H, alkyl chain), 1.31–1.11 (m, 8H, alkyl chain), 1.08 (t, *J* = 7.1 Hz, 3H, OCH_2_C*H*_3_), 0.79 (m, 6H, alkyl chain). ^13^C NMR (101 MHz, DMSO) *δ* 164.7, 162.4, 140.6, 128.9, 127.4, 126.8, 111.5, 60.0, 56.5, 44.0, 30.4, 28.6, 28.0, 24.0, 23.6, 22.9, 22.7, 14.5, 14.3. HRMS (TOF MS ASAP+): calculated for C_19_H_28_NO_4_S^+^: 366.1739. Found *m*/*z* 366.1739 [M + H]^+^.

### Ethyl-1-dodecyl-4-hydroxy-5-oxo-2-(thiophen-2-yl)pyrrolidine-3-carboxylate 3a

To an oven dried flask under argon was added enol 2a, (15 g, 35.60 mmol), zinc powder (13.80 g, 212 mmol) and a few drops of H_2_SO_4_ into acetic acid (500 mL). The reaction was heated to 100 °C and allowed to stir for 2 h. This then followed by the addition of a second portion of zinc (13.80 g, 212 mmol) and the reaction was stirred for a further 1 h. The reaction mixture was then cooled to RT and the excess zinc/inorganic salts were removed *via* filtration. The filtrate was diluted with 300 mL of water and the organic layer was extracted with dichloromethane. The combined organic layer was then washed with saturated NaHCO_3_ solution until neutral and dried over MgSO_4_.The solution was then concentrated *in vacuo* to give a crude oil. Hexane was added to the oil and the white precipitate that formed was collected *via* filtration and washed with hexane to afford the product as a crude white solid (9.02 g, 66%), which was used in the next reaction without further purification. HRMS (TOF MS ASAP+): calculated for C_23_H_38_NO_4_S^+^: 424.2522. Found *m*/*z* 424.2511 [M + H]^+^.

### Ethyl-1-(2-ethylhexyl)-4-hydroxy-5-oxo-2-(thiophen-2-yl)pyrrolidine-3-carboxylate 3b

Compound 3b was made using the same procedure as making 3a with the following reagents: enol 2b (16.65 g, 47 mmol), zinc powder (18.32 g, 280 mmol) and a few drops of sulfuric acid in acetic acid (500 mL). The combined organic layer was then washed with saturated NaHCO_3_ solution until neutral and dried over MgSO_4_.The solution was then concentrated *in vacuo* to afford the product as a crude red oil (12.98 g, 78%), which was used in the next reaction without further purification. HRMS (TOF MS ASAP+): calculated for C_19_H_30_NO_4_S^+^: 368.1896. Found *m*/*z* 368.1907 [M + H]^+^.

### Ethyl-1-dodecyl-5-oxo-2-(thiophen-2-yl)-4,5-dihydro-1*H*-pyrrole-3-carboxylate 4a

To an oven dried flask under argon equipped with a reflux condenser was added alcohol 3a, (9.02 g, 20 mmol), mesyl chloride (2.68 g, 23 mmol), and TEA portion wise (8.61 g, 85 mmol). The reaction mixture was then heated under reflux and allowed to stir for 30 minutes. After cooling to RT, the mixture was diluted with 5% HCl solution (100 mL). The aqueous layers were extracted with dichloromethane and the combined organic layers were washed with 5% HCl solution followed by sat Na_2_HCO_3_ until neutral, and finally water. The solution was dried over MgSO_4_ concentrated *in vacuo* to give the crude brown residue which was purified *via* dissolving in minimum MeOH and freezing overnight. The solid was collected *via* filtration to afford the product as a mink crystals (4.02 g, 9.9 mmol, 49%). ^1^H NMR (400 MHz, CDCl_3_) *δ* 7.53 (dd, *J* = 5.0, 1.1 Hz, 1H, ThH), 7.16 (dd, *J* = 3.5, 1.1 Hz, 1H, ThH), 7.13 (dd, *J* = 5.0, 3.5 Hz, 1H, ThH), 4.05 (q, *J* = 7.1 Hz, 2H, OC*H*_2_CH_3_), 3.44 (s, 2H, NCOCH_2_), 3.42–3.37 (m, 2H, NCH_2_), 1.40 (m, 2H, alkyl chain), 1.32–1.12 (m, 18H, alkyl chain), 1.10 (t, *J* = 7.1 Hz, 3H, OCH_2_C*H*_3_), 0.87 (t, *J* = 6.9 Hz, 3H, alkyl chain). ^13^C NMR (101 MHz, CDCl_3_) *δ* 175.4, 162.8, 147.9, 130.0, 128.8, 128.4, 127.0, 108.3, 59.9, 41.1, 37.5, 31.9, 29.6, 29.5, 29.4, 29.3, 29.0, 28.9, 26.6, 22.7, 14.1, 14.0. HRMS (TOF MS ASAP+): calculated for C_23_H_36_NO_3_S^+^: 406.2416. Found *m*/*z* 406.2416 [M + H]^+^.

### Ethyl-1-(2-ethylhexyl)-5-oxo-2-(thiophen-2-yl)-4,5-dihydro-1*H*-pyrrole-3-carboxylate 4b

Compound 4b was made using the same procedure as making 4a with the following reagents, alcohol 3b (1.16 g, 3.16 mmol) dissolved in anhydrous chloroform (20 mL), mesyl chloride (0.39 g, 3.50 mmol) and TEA (1.27 g, 12.6 mmol). The crude brown residue, which was purified *via* column chromatography on silica gel (chloroform : ethyl acetate = 95 : 5), to afford the pure product as a light brown/yellow oil (0.35 g, 1.00 mmol, 32%). ^1^H NMR (400 MHz, CDCl_3_) *δ* 7.53 (dd, *J* = 4.9, 1.3 Hz, 1H, ThH), 7.15 (dd, *J* = 3.6, 1.3 Hz, 1H, ThH), 7.12 (dd, *J* = 4.9, 3.6 Hz, 1H, ThH), 4.06 (q, *J* = 7.1 Hz, 2H, OC*H*_2_CH_3_), 3.46 (s, 2H, NCOCH_2_), 3.44–3.31 (m, 2H, NCH_2_), 1.31–1.24 (m, 1H, alkyl chain), 1.11 (t, *J* = 7.1 Hz, 3H, OCH_2_C*H*_3_), 1.22–1.00 (m, 8H, alkyl chain), 0.82 (t, *J* = 7.2 Hz, 3H, alkyl chain), 0.69 (t, *J* = 7.4 Hz, 3H, alkyl chain). ^13^C NMR (101 MHz, CDCl_3_) *δ* 175.7, 162.8, 148.0, 130.0, 128.9, 128.3, 126.9, 108.4, 60.0, 44.6, 38.6, 37.4, 30.4, 28.4, 23.8, 22.8, 14.0, 14.0, 10.5. HRMS (TOF MS ASAP+): calculated for C_19_H_28_NO_3_S^+^: 350.1790. Found *m*/*z* 350.1793 [M + H]^+^.

### 6-(4-Bromophenyl)-2-dodecyl-3-(thiophen-2-yl)-2,5-dihydropyrrolo[3,4-*c*]pyrrole-1,4-dione 5ad

To an oven dried flask under argon set up for reflux, was added sodium chunks (0.46 g, 19.90 mmol) and 2-methyl-2-butanol (26.00 mL), followed by anhydrous iron(iii) chloride (0.01 g). The mixture was allowed to stir at reflux until all sodium had been consumed. The reaction was then cooled to 95 °C and 4a (2.60 g, 6.40 mmol) was added to the reaction mixture, followed by 4-bromobenzonitrile (1.40 g, 7.69 mmol) after 1 minute. The reaction turned red in colour and was left to stir at 95 °C overnight. The mixture was then allowed to cool to 50 °C, followed by the addition of MeOH (35.00 mL). The reaction was quenched with glacial acetic acid (4.00 mL) and was cooled to RT. The solid that precipitated out of solution was washed with MeOH and collected *via* filtration to yield the product as a pink solid (1.01 g, 1.86 mmol, 28%). ^1^H NMR (400 MHz, CDCl_3_) *δ* 9.85 (br s, 1H, NH), 8.95 (dd, *J* = 4.0, 1.1 Hz, 1H, ThH), 8.21 (d, *J* = 8.6 Hz, 2H, PhH), 7.71 (dd, *J* = 5.0, 1.1 Hz, 1H, ThH), 7.58 (d, *J* = 8.6 Hz, 2H, PhH), 7.29 (dd, *J* = 5.0, 4.0 Hz, 1H, ThH), 4.12–3.96 (m, 2H, NCH_2_), 1.81–1.69 (m, 2H, alkyl chain), 1.48–1.21 (m, 20H, alkyl chain), 0.88 (t, *J* = 6.8 Hz, 3H, alkyl chain). ^13^C NMR (101 MHz, CDCl_3_) *δ* 162.4, 161.8, 141.9, 141.6, 136.2, 132.3, 131.7, 129.5, 129.0, 128.6, 126.6, 126.1, 110.1, 108.7, 42.4, 31.9, 29.9, 29.6, 29.5, 29.4, 29.3, 26.9, 22.7, 14.1. HRMS (TOF MS ASAP+): calculated for C_28_H_34_BrN_2_O_2_S^+^: 541.1524. Found *m*/*z* 541.1534 [M + H]^+^.

### 2-(2-Ethylhexyl)-6-phenyl-3-(thiophen-2-yl)-2,5-dihydropyrrolo[3,4-*c*]pyrrole-1,4-dione 5bc

To an oven dried flask under argon set up for reflux, was added sodium chunks (0.46 g, 19.92 mmol) and 2-methyl-2-butanol (27.00 mL), followed by anhydrous iron(iii) chloride (0.01 g). The mixture was allowed to stir at reflux until all sodium had been consumed (30 min). The reaction was then cooled to 95 °C and 4b (2.00 g, 5.72 mmol) was added to the reaction mixture, followed by benzonitrile (0.71 g, 6.87 mmol) after 1 minute. The reaction turned red in colour and was left to stir at 95 °C overnight. The mixture was then allowed to cool to 50 °C, followed by the addition of MeOH (27.00 mL). The reaction was quenched with glacial acetic acid (6.00 mL) and cooled to RT. The solid that precipitated out of solution was washed with MeOH and collected *via* filtration to yield the product as a pink solid (0.40 g, 0.98 mmol, 17%). ^1^H NMR (600 MHz, CDCl_3_) *δ* 10.05 (s, 1H, NH), 8.97 (dd, *J* = 3.9, 0.8 Hz, 1H, ThH), 8.41 (d, *J* = 6.8 Hz, 2H, PhH), 7.69 (dd, *J* = 5.0, 0.8 Hz, 1H, ThH), 7.56–7.47 (m, 3H, PhH), 7.32 (dd, *J* = 5.0, 3.9 Hz, 1H, ThH), 4.08–4.00 (m, 2H, NCH_2_), 1.92–1.84 (m, 1H, NCH_2_C*H*), 1.43–1.18 (m, 8H, alkyl chain), 0.87 (m, 6H, alkyl chain). ^13^C NMR (151 MHz, CDCl_3_) *δ* 162.8, 162.3, 143.3, 141.8, 135.9, 131.7, 131.2, 129.7, 129.1, 128.3, 127.9, 109.5, 109.3, 46.0, 39.1, 30.2, 28.4, 23.5, 23.1, 14.0, 10.5. HRMS (TOF MS ASAP+): calculated for C_24_H_27_N_2_O_2_S^+^: 407.1793. Found *m*/*z* 407.1775 [M + H]^+^.

### 6-(4-Bromophenyl)-2-(2-ethylhexyl)-3-(thiophen-2-yl)-2,5-dihydropyrrolo[3,4-*c*]pyrrole-1,4-dione 5bd

To an oven dried flask under argon set up for reflux, was added sodium chunks (0.736 g, 32.01 mmol) and 2-methyl-2-butanol (42.00 mL), followed by anhydrous iron(iii) chloride (0.01 g). The mixture was allowed to stir at reflux until all sodium had been consumed (30 min). The reaction was then cooled to 95 °C and 4b (3.60 g, 10.30 mmol) was added to the mixture, followed by 4-bromobenzonitrile (0.32 g, 3.43 mmol) after 1 minute. The reaction turned red in colour and was left to stir at 95 °C overnight. The mixture was then allowed to cool to 50 °C, followed by the addition of MeOH (40.00 mL). The reaction was quenched with glacial acetic acid (4.00 mL). The reaction mixture was then cooled to RT followed by the addition of water (100 mL). The organic layer was then extracted with ethyl acetate × 3 and dried over MgSO_4_. The resulting solution was filtered and concentrated *in vacuo* to afford a red solid, which was partially purified *via* washing with MeOH and collected *via* filtration to yield the product as a dark pink solid (0.142 g, 0.16 mmol, 3%) which was used in the next reaction without further purification. ^1^H NMR (400 MHz, CDCl_3_) *δ* 10.02 (s, 1H, NH), 8.95 (dd, *J* = 3.9, 0.8 Hz, 1H, ThH), 8.26 (d, *J* = 8.7 Hz, 2H, PhH), 7.72 (dd, *J* = 5.0, 0.8 Hz, 1H, ThH), 7.61 (d, *J* = 8.7 Hz, 2H, PhH), 7.31 (dd, *J* = 5.0, 3.9 Hz, 1H, ThH), 4.07–3.97 (m, 2H, NCH_2_), 1.86 (m, 1H, alkyl chain), 1.40–1.20 (m, 8H, alkyl chain), 0.87 (dt, *J* = 11.0, 7.3 Hz, 6H, alkyl chain). ^13^C NMR (101 MHz, CDCl_3_) *δ* 162.5, 162.2, 142.3, 141.7, 136.2, 132.4, 131.7, 129.6, 129.1, 128.4, 126.7, 126.1, 110.0, 109.1, 46.0, 39.1, 30.2, 28.3, 23.5, 23.1, 14.0, 10.5. HRMS (TOF MS ASAP+): calculated for C_24_H_26_BrN_2_O_2_S^+^: 485.0898. Found *m*/*z* 485.0891 [M + H]^+^.

### 2-(2-Ethylhexyl)-6-(furan-2-yl)-3-(thiophen-2-yl)-2,5-dihydropyrrolo[3,4-*c*]pyrrole-1,4-dione 5be

To an oven dried flask under argon set up for reflux, was added sodium chunks (0.46 g, 20.00 mmol) and 2-methyl-2-butanol (27.20 mL), followed by anhydrous iron(iii) chloride (0.01 g). The mixture was allowed to stir at reflux until all sodium had been consumed. The reaction was then heated to 120 °C and 4b (2.00 g, 5.72 mmol) was added to the reaction mixture, followed by 2-furanitrile (0.60 g, 6.45 mmol). The reaction turned red in colour and was left to stir at 120 °C for 4 h. The mixture was then allowed to cool to 50 °C, followed by the addition of MeOH (27.20 mL). The reaction was quenched with glacial acetic acid (6.80 mL). The reaction mixture was then cooled to RT followed by the addition of water (200 mL). The organic layer was then extracted with ethyl acetate × 3 and dried over MgSO_4_. The resulting solution was filtered and concentrated *in vacuo* to afford a crude red oil, which was partially purified *via* column chromatography on silica gel (chloroform : ethyl acetate = 80 : 20), to give the product as a dark pink oil (0.240 g, 0.61 mmol, 10%) which was used in the next reaction without further purification. ^1^H NMR (400 MHz, CDCl_3_) *δ* 8.80 (d, *J* = 3.5 Hz, 1H, ArH), 8.06 (br s, 1H, NH), 7.88 (d, *J* = 3.5 Hz, 1H, ArH), 7.64 (m, 1H, ArH), 7.60 (m, 1H, ArH), 7.31–7.24 (m, 1H, ArH), 6.68 (d, *J* = 2.1 Hz, 1H, ArH), 4.03–4.00 (m, 2H, NCH_2_), 1.89–1.81 (m, 1H, alkyl chain), 1.43–1.16 (m, 8H, alkyl chain), 0.87 (m, 6H, alkyl chain). ^13^C NMR (101 MHz, CDCl_3_) *δ* 161.3, 161.2, 145.4, 143.6, 140.9, 135.4, 130.8, 129.8, 128.5, 118.3, 113.9, 108.0, 107.3, 45.8, 39.1, 30.2, 28.3, 23.5, 23.1, 14.0, 10.5. HRMS (TOF MS ASAP+): calculated for C_22_H_25_N_2_O_3_S^+^: 397.1586. Found *m*/*z* 397.1596 [M + H]^+^.

### 3-(4-Bromophenyl)-5-dodecyl-2-(2-ethylhexyl)-6-(thiophen-2-yl)-2,5-dihydropyrrolo[3,4-*c*]pyrrole-1,4-dione 6adh

To an oven dried vial under argon was added 5ad (0.20 g, 0.37 mmol), K_2_CO_3_ (0.086 g, 0.63 mmol), 18-crown-6 (5 mg), DMF (4.40 mL) and 2-ethylhexyl bromide (0.120 g, 0.63 mmol). The vial was sealed, and the reaction was stirred for 2 nights at 120 °C. The reaction mixture was then cooled to RT. Chloroform was then added to the flask and the solution was concentrated *in vacuo*. The crude product was then purified *via* column chromatography on silica gel (hexane : chloroform = 2 : 1) to afford the product as an orange/pink oil (0.036 g, 0.06 mmol, 15%). ^1^H NMR (400 MHz, CDCl_3_) *δ* 8.97 (dd, *J* = 4.0, 1.0 Hz, 1H, ThH), 7.71–7.60 (m, 4H, 1H, PhH, ThH), 7.29 (dd, *J* = 4.9, 4.0 Hz, 1H, ThH), 4.03–3.95 (m, 2H, NCH_2_), 3.85–3.72 (m, 2H, NCH_2_), 1.71 (m, 2H, alkyl chain), 1.56–1.02 (m, 27H, alkyl chain), 0.87 (t, *J* = 6.8 Hz, 3H, CH_3_), 0.80 (t, *J* = 6.8 Hz, 3H, CH_3_), 0.72 (t, *J* = 7.4 Hz, 3H, CH_3_). ^13^C NMR (100 MHz, CDCl_3_) *δ* 162.0, 161.9, 145.2, 142.2, 136.0, 132.1, 131.3, 130.1, 129.6, 128.7, 127.5, 125.2, 110.1, 107.4, 45.1, 42.1, 38.6, 31.9, 30.3, 29.9, 29.6, 29.5, 29.3, 29.2, 28.2, 26.8, 23.7, 22.9, 22.7, 14.1, 14.0, 10.4. HRMS (TOF MS ASAP+): calculated for C_36_H_50_BrN_2_O_2_S^+^: 653.2776. Found *m*/*z* 653.2781 [M + H]^+^.

### 3-(4-Bromophenyl)-5-dodecyl-6-(thiophen-2-yl)-2-(2,5,8,11-tetraoxatridecan-13-yl)-2,5-dihydropyrrolo[3,4-*c*]pyrrole-1,4-dione 6adi

Compound 6adi was made using the same procedure as making 6adh with the following reagents, 5ad (0.171 g, 0.32 mmol), K_2_CO_3_ (0.075 g, 0.54 mmol), 18-crown-6 (12 mg), DMF (3.80 mL) and triethylene glycol 2-bromoethyl methyl ether (0.14 g, 0.54 mmol). The crude product was then purified *via* column chromatography on silica gel (hexane : chloroform = 2 : 1) followed by (chloroform : ethyl acetate = 80 : 20) to afford the product as an orange oil (0.012 g, 0.016 mmol, 5%). ^1^H NMR (400 MHz, CDCl_3_) *δ* 8.91 (d, *J* = 3.9 Hz, 1H, ThH), 7.91 (d, *J* = 8.5 Hz, 2H, PhH), 7.68 (d, *J* = 3.9 Hz, 1H, ThH), 7.64 (d, *J* = 8.5 Hz, 2H, PhH), 7.32–7.28 (m, 1H, ThH), 4.02–3.93 (m, 4H, NCH_2_CH_2_O), 3.79 (t, *J* = 5.3 Hz, 2H, NCH_2_), 3.63–3.49 (m, 12H, OCH_2_CH_2_O), 3.35 (s, 3H, OCH_3_), 1.70 (m, 2H, alkyl chain), 1.43–1.20 (m, 18H, alkyl chain), 0.87 (t, *J* = 6.8 Hz, 3H, alkyl chain). ^13^C NMR (100 MHz, CDCl_3_) *δ* 162.2, 161.9, 145.9, 142.1, 135.8, 132.0, 131.4, 131.0, 129.6, 128.7, 126.9, 125.5, 109.9, 107.3, 71.9, 70.6, 70.5, 69.0, 59.0, 42.5, 42.1, 31.9, 29.9, 29.6, 29.5, 29.3, 29.2, 26.9, 22.7, 14.1. HRMS (TOF MS ASAP+): calculated for C_37_H_52_BrN_2_O_6_S^+^: 731.3729. Found *m*/*z* 731.3709 [M + H]^+^.

### 3-(4-Bromophenyl)-2-dodecyl-5-(2-ethylhexyl)-6-(thiophen-2-yl)-2,5-dihydropyrrolo[3,4-*c*]pyrrole-1,4-dione 6bdf

Compound 6bdf was made using the same procedure as making 6adh with the following reagents, 5bd (0.14 g, 0.29 mmol), K_2_CO_3_ (0.073 g, 0.53 mmol), 18-crown-6 (5 mg), DMF (3.40 mL) and 1-bromododecane (0.13 g, 0.53 mmol). The crude product was then purified *via* column chromatography on silica gel (hexane : chloroform = 2 : 1) to afford the product as an orange/pink oil (0.030 g, 0.05 mmol, 16%). ^1^H NMR (600 MHz, CDCl_3_) *δ* 8.91 (dd, *J* = 3.9, 1.1 Hz, 1H, ThH), 7.70–7.63 (m, 4H, 1H, PhH, ThH), 7.28 (dd, *J* = 5.0, 3.9 Hz, 1H, ThH), 3.95 (m, 2H, NCH_2_), 3.81–3.75 (m, 2H, NCH_2_), 1.85–1.78 (m, 1H, NCH_2_C*H*), 1.65–1.58 (m, 2H, NCH_2_C*H*_2_), 1.38–1.18 (m, 26H, alkyl chain), 0.86 (m, 9H, CH_3_). ^13^C NMR (100 MHz, CDCl_3_) *δ* 162.3, 161.9, 145.2, 142.4, 135.8, 132.2, 131.2, 130.1, 129.6, 128.5, 127.2, 125.4, 109.7, 107.9, 45.7, 42.1, 39.1, 30.2, 29.6, 29.5, 29.3, 29.1, 28.4, 26.7, 23.5, 23.1, 22.7, 14.1, 14.0, 10.5. HRMS (TOF MS ASAP+): calculated for C_36_H_49_O_2_N_2_BrNaS^+^: 675.2590. Found *m*/*z* 675.2601 [M + Na]^+^.

### 2-(2-Ethylhexyl)-6-(furan-2-yl)-5-(2-octyldodecyl)-3-(thiophen-2-yl)-2,5-dihydropyrrolo[3,4-*c*]pyrrole-1,4-dione 6beg

Compound 6beg was made using the same procedure as making 6adh with the following reagents, 5be (0.17 g, 0.42 mmol), K_2_CO_3_ (0.98 g, 0.71 mmol), 18-crown-6 (0.012 g), DMF (12 mL) and 9-(bromomethyl)nonadecane (0.26 g, 0.71 mmol). The crude product was then purified *via* column chromatography on silica gel (hexane : chloroform = 2 : 1) to afford the product as a pink oil (0.082 g, 0.12 mmol, 29%). ^1^H NMR (400 MHz, CDCl_3_) *δ* 8.89 (d, *J* = 3.8 Hz, 1H, ArH), 8.36 (d, *J* = 3.8 Hz, 1H, ArH), 7.61 (d, *J* = 1.9 Hz, 2H, ArH), 7.28–7.24 (m, 1H, ArH), 6.69 (dd, *J* = 3.8, 1.6 Hz, 1H, ArH), 4.03 (m, 4H, NCH_2_), 1.75–1.71 (m, 2H, alkyl chain), 1.42–1.05 (m, 40H, alkyl chain), 0.86 (m, 12H, alkyl chain). ^13^C NMR (101 MHz, CDCl_3_) *δ* 161.7, 161.3, 144.9, 144.6, 140.2, 135.2, 134.2, 130.3, 129.9, 128.4, 120.3, 113.5, 108.0, 46.6, 45.8, 42.0, 39.1, 38.5, 31.9, 31.5, 30.2, 30.0, 29.6, 29.3, 28.3, 27.0, 26.5, 25.0, 23.5, 23.1, 22.7, 14.1, 14.0, 10.5. HRMS (TOF MS ASAP+): calculated for C_42_H_65_N_2_O_3_S^+^: 677.4716. Found *m*/*z* 677.4720 [M + H]^+^.

## Conflicts of interest

There are no conflicts to declare.

## Supplementary Material

RA-011-D0RA01564D-s001
