# Supplementary material for: Synthesis of fully asymmetric diketopyrrolopyrrole derivatives
Source: RSC Adv. 2021 Jan 27;11(9):5276–83. doi: 10.1039/d0ra01564d (PMC8694678; doi:10.1039/d0ra01564d)
Supplement: RA-011-D0RA01564D-s001 [file RA-011-D0RA01564D-s001.pdf]

Electronic Supplementary Material (ESI) for RSC Advances.
This journal is © The Royal Society of Chemistry 2020

Supporting information

Synthesis of Fully Asymmetric Diketopyrrolopyrrole Derivatives

Lisa Sharma,[a] Hugo Bronstein [b*]

[1] Department of Chemistry, University of Cambridge, Lensfield Rd, Cambridge CB2 1EW

Contents

1. General Experimental
2. Optimization of **5ad**
3. ^{1}H NMR and ^{13}C NMR Spectra
4. DFT Calculations
5. References

1. General Experimental

General Experimental

All moisture and air sensitive reactions were carried out in oven dried flasks under an inert argon atmosphere. R.T refers to 25˚C maintained by the use of a heating mantle. All reactions were covered with foil unless otherwise stated and were magnetically stirred. Merck Geduran® Si 60 silica gel or Biotage® Isolera™ Four with either Biotage® SNAP/ SNAP Ultra cartridges (10 g, 20 g, 50 g or 100 g) were used for column chromatography during purification. DC Fertigfolien ALUGRAM aluminium sheets coated with silica gel, were used for carrying out analytical thin layer chromatography (TLC). Compounds were visualised by ultra-violet light. Chemicals were used as supplied. Anhydrous solvents were supplied commercially and used under an inert argon atmosphere. All other solvents and reagents used were supplied commercially and used as received.

Instrumental Techniques

^{1}H NMR were carried out at 400 MHz on an Avance III 400 HD Spectrometer or at 600 MHz on an Avance 600 BBI Spectrometer at the Department of Chemistry, University of Cambridge. The internal standard used was $CHCl_3$ (δ = 7.26 ppm s) and DMSO (δ = 2.50 ppm, s). ^{1}H NMR shifts were reported to the nearest 0.01 ppm and the following abbreviations were used: s, singlet; d, doublet; t, triplet; q, quartet; qn, quintet; sxt, sextet; m, multiplet; br, broad; Ar, aromatic; Th, thienyl; Ph, phenyl. The coupling constants (*J*) are measured in Hertz. ^{13}C NMR spectra were recorded at 125 MHz on a BRUKER DCH Cryoprobe Spectrometer in the stated solvent. The internal standard used was ^{13}C NMR (δ = 77.2 ppm, t) and $(CD_3)_2SO$ (δ = 39.52 ppm, s). ^{13}C NMR chemical shifts are reported to the nearest 0.1 ppm. Mass spectra were obtained using a Waters LCT, Finnigan MAT 900XP or Waters MALDI micro MX spectrometer at the Department of Chemistry, University of Cambridge. UV-vis spectra were recorded on a Shimadzu UV-1800 spectrophotometer using Hellma® absorption cuvettes in chlorobenzene (~4 μg/mL), 200-2500 nm spectral range, pathlength 10 mm, chamber volume 3500 μL. The molecular molar extinction coefficient (ε) was calculated according to the Beer Lambert Law $A = \varepsilon \cdot l \cdot c$.

2. Optimization of 5ad

Optimized reaction conditions for the synthesis of **5ad** were investigated and are summarised in **Table**

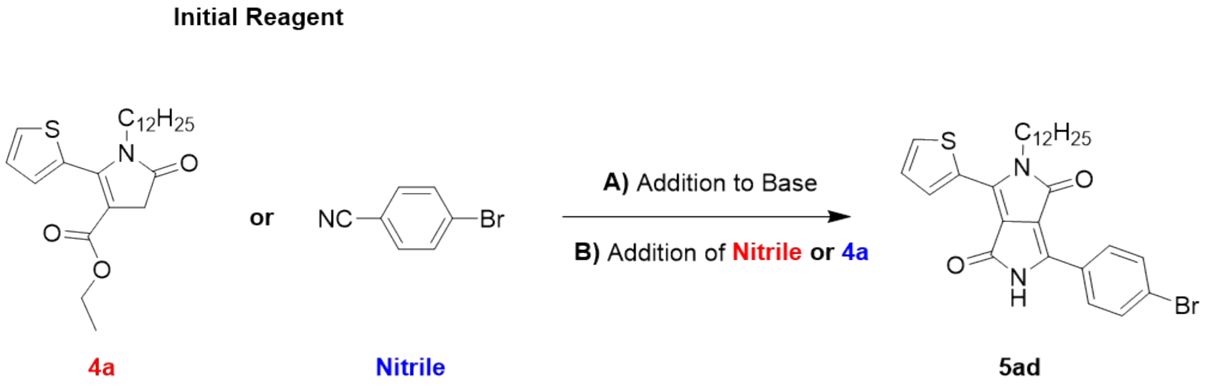

S1.

Entry	Initial Reagent	Step A (min)	Step B (h)	Reaction Conditions	Reaction Temperature (°C)	Yield (%)
1	**4a**	1	2	Na, TAA	95	Trace
2	**4a**	1	4	Na, TAA	95	3
3	**4a**	1	18	Na, TAA	95	28
4	**4a**	1	18	Na, TAA	120	8
5	**4a**	10	18	Na, TAA	95	16
6	**4a**	30	18	Na, TAA	95	Trace
7	**4a**	60	18	Na, TAA	95	Trace
8	**4a**	1	18	Pyrrolidine, DCM	50	Trace
9	**4a**	15	18	NaH, DMSO	R.T	Trace
10	**4a**	15	18	NaH, DMSO	95	Trace
11	**Nitrile**	1	18	Na, TAA	95	9
12	**Nitrile**	10	18	Na, TAA	95	0.4
13	**Nitrile**	60	18	Na, TAA	95	Trace

Table S1.- Summary of optimization attempts for the synthesis of **5ad**. Reactions were conducted in the presence of 1 equiv. of **4a**, 1.2 equiv. of nitrile (4-bromobenzonitrile) and 3.1 equiv. of Na in TAA (tert-Amyl alcohol/2-methyl-2-butanol). Yields have been calculated on isolated and purified compounds.

The ´Initial Reagent´ refers to the order of addition of the coupling reagents **4a** and **4-bromobenzonitrile,** following the formation of the base**.** In an attempt to improve the yield, it was thought that a longer deprotonation time may be required (**Step A**), before addition of the second coupling reagent (**Step B**) in order to drive the reaction to completion. However, upon investigation, a shorter deprotonation time was found to be the most effective. It was also observed that beyond a

deprotonation time of 10 minutes, degradation of **4a** could be observed by ^{1}H NMR. Alternative bases to NaOt-Am, such as NaH and pyrrolidine were also tested, however only trace amount of product was detected *via* TLC and ^{1}H NMR

3. ^{1}H NMR and ^{13}C NMR Spectra

^{1}H NMR Spectra for **2a**

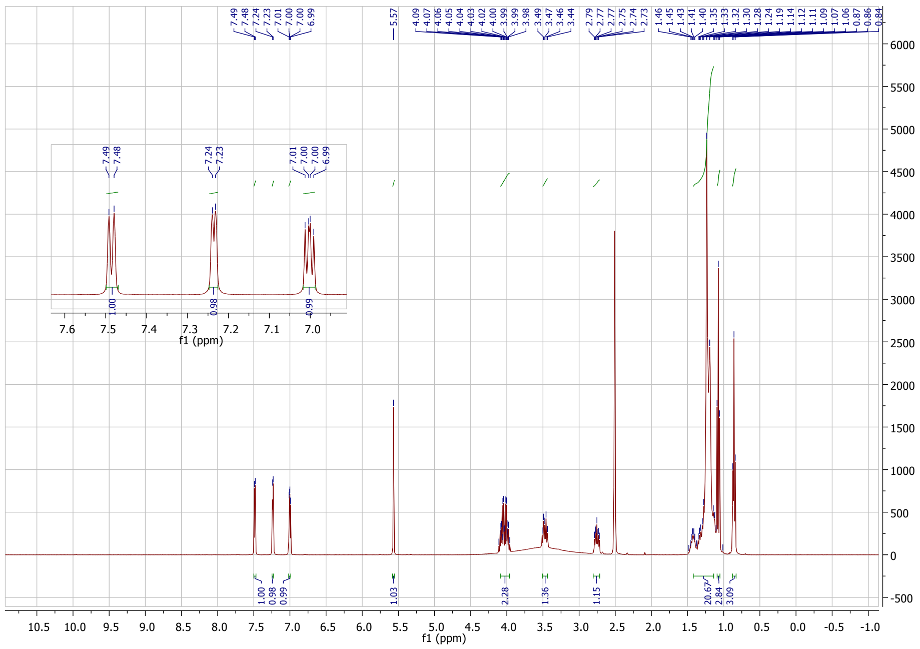

^{13}C NMR Spectra for **2a**

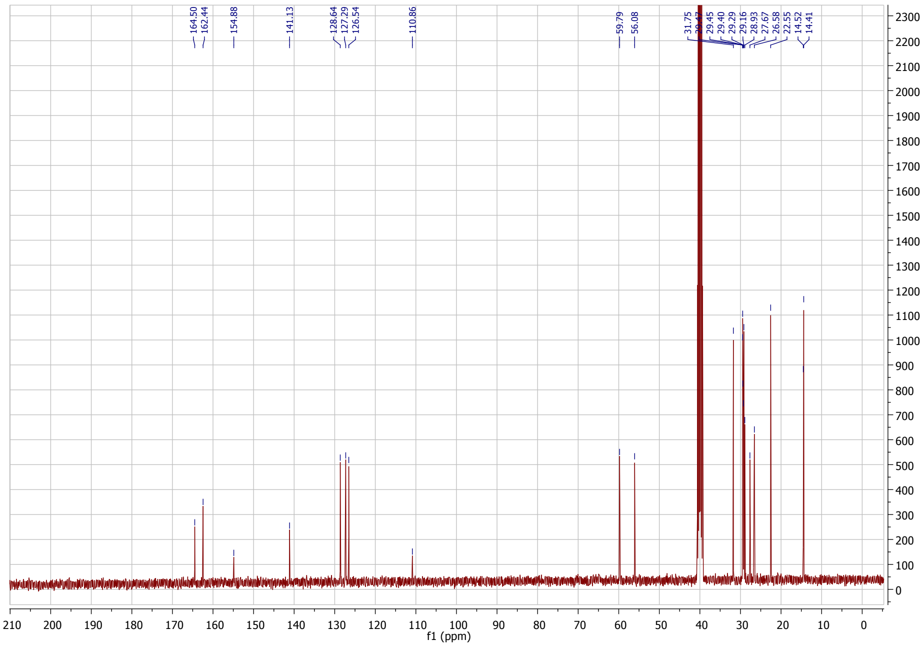

^{1}H NMR Spectra for **2b**

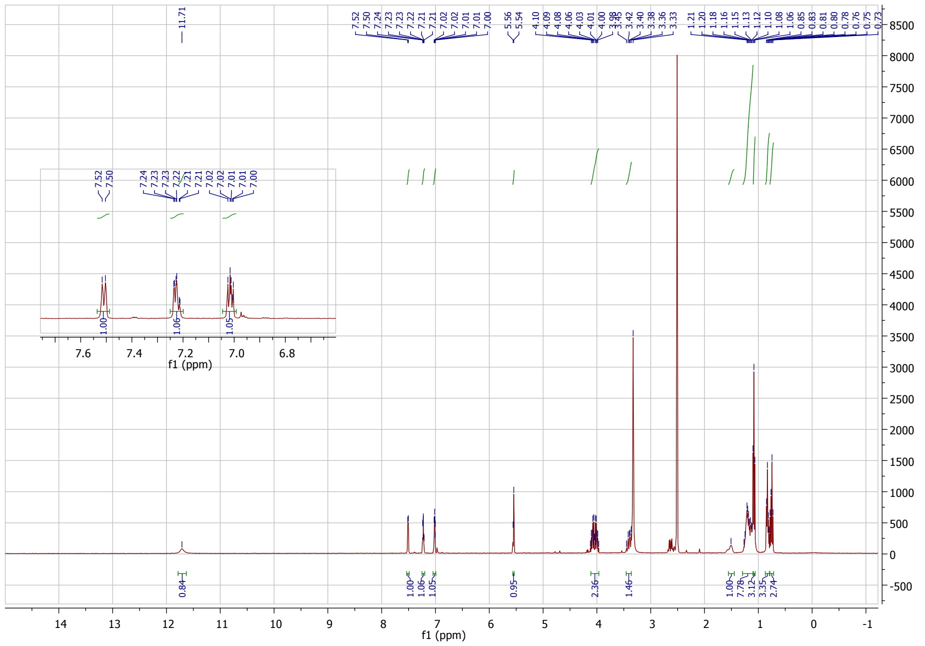

^{13}C NMR Spectra for **2b**

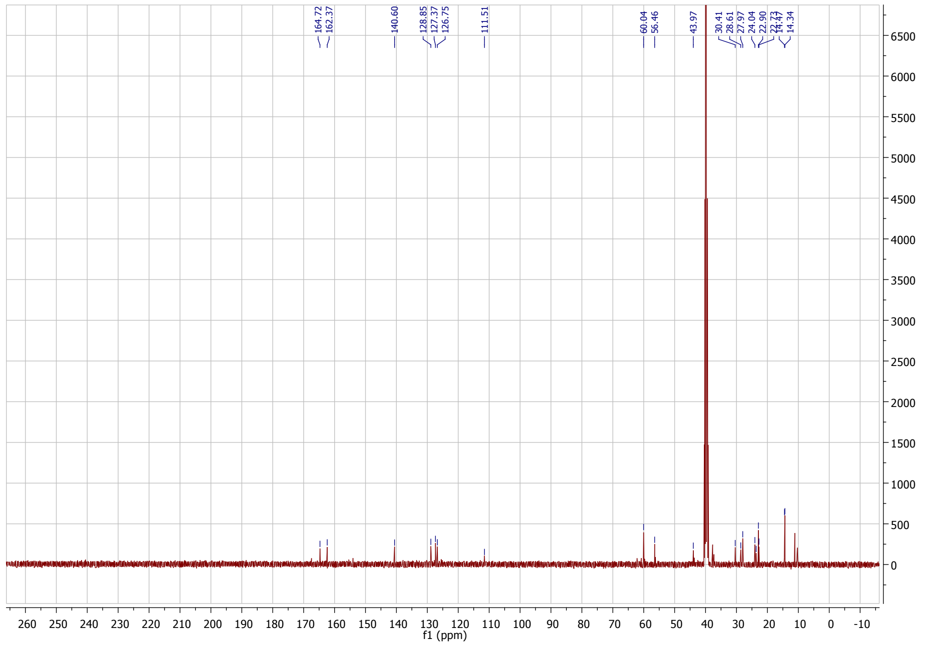

^{1}H NMR Spectra for **4a**

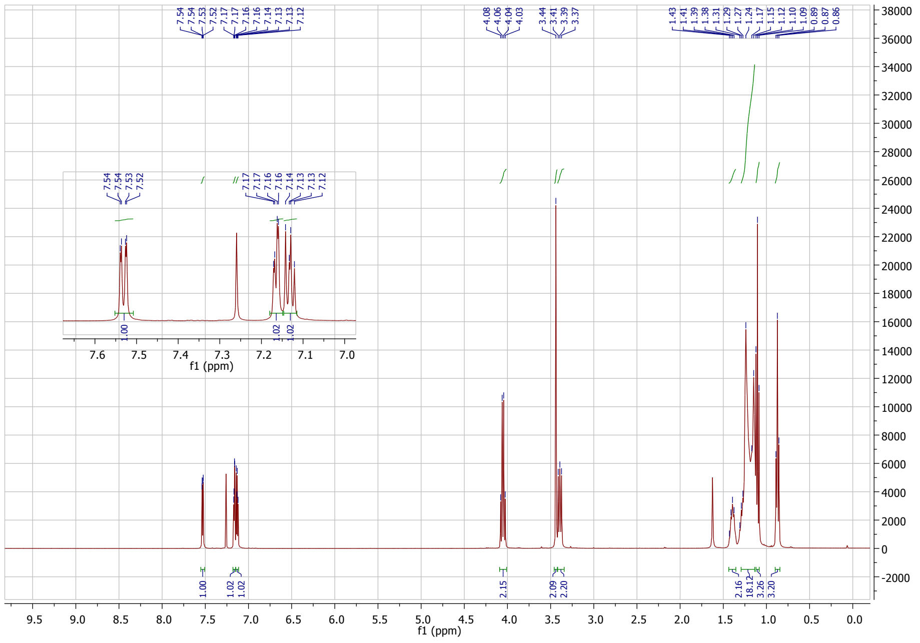

^{13}C NMR Spectra for **4a**

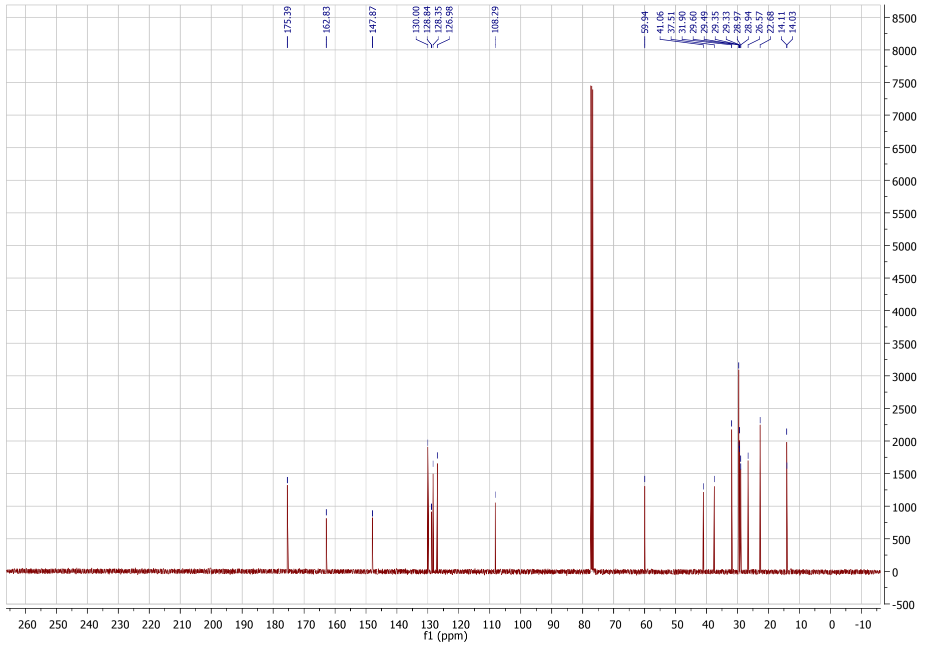

^{1}H NMR Spectra for **4b**

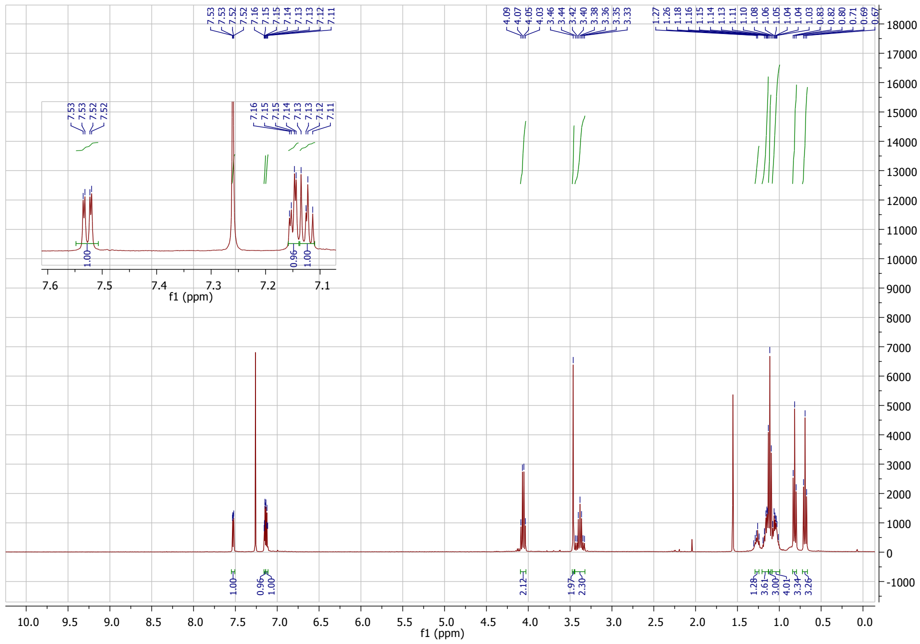

^{13}C NMR Spectra for **4b**

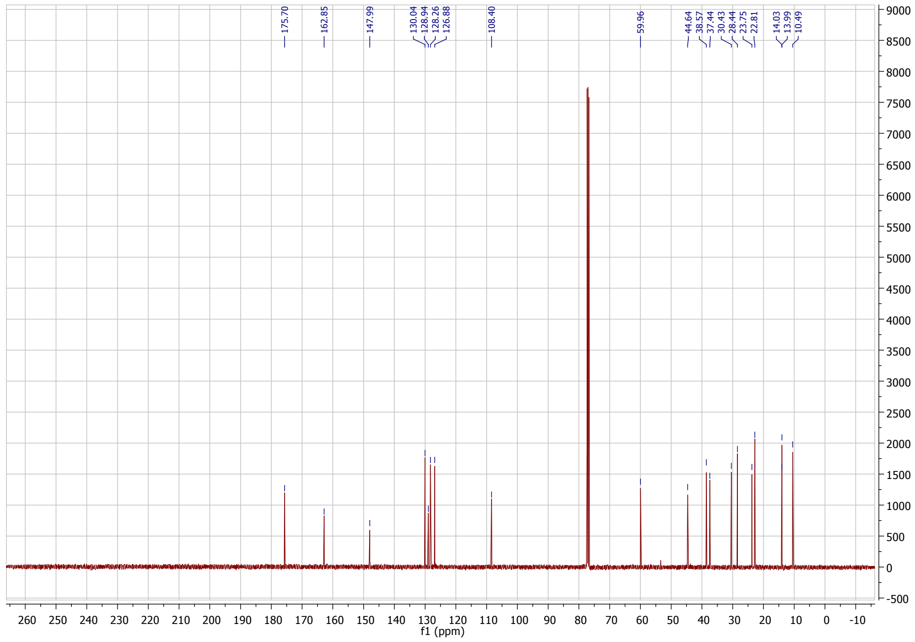

^{1}H NMR Spectra for **5ad**

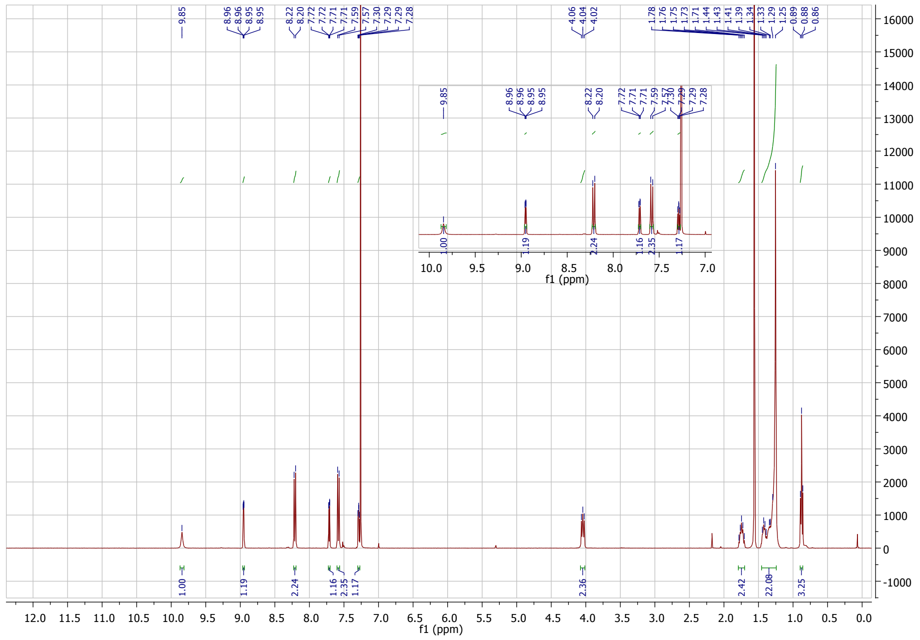

^{13}C NMR Spectra for **5ad**

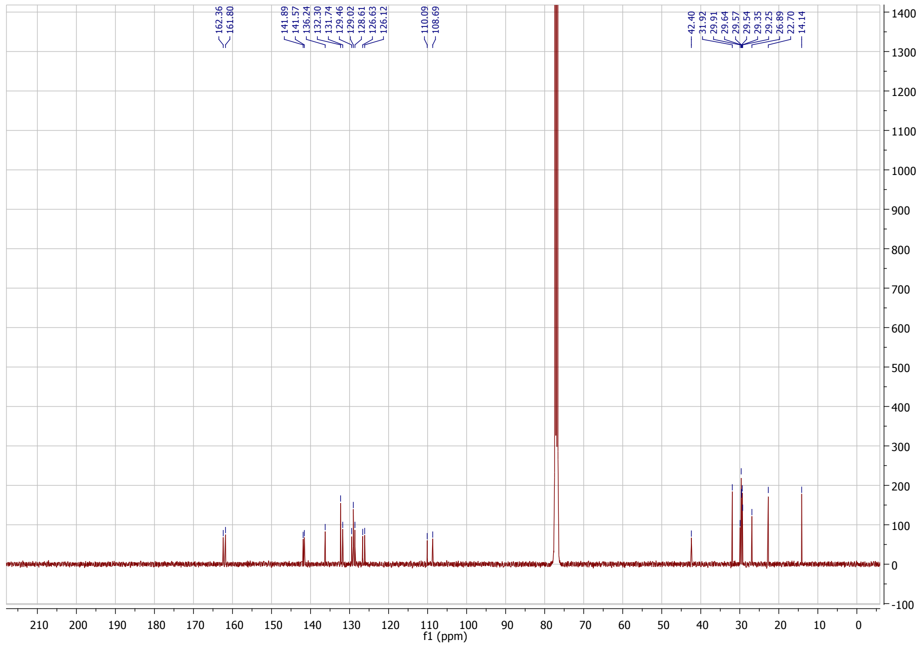

^{1}H NMR Spectra for **5bc**

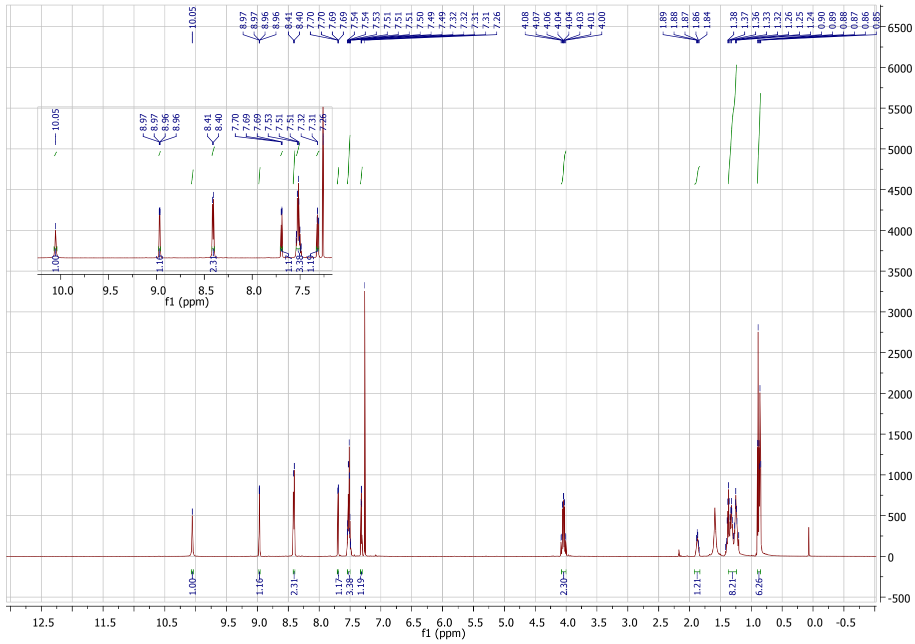

^{13}C NMR Spectra for **5bc**

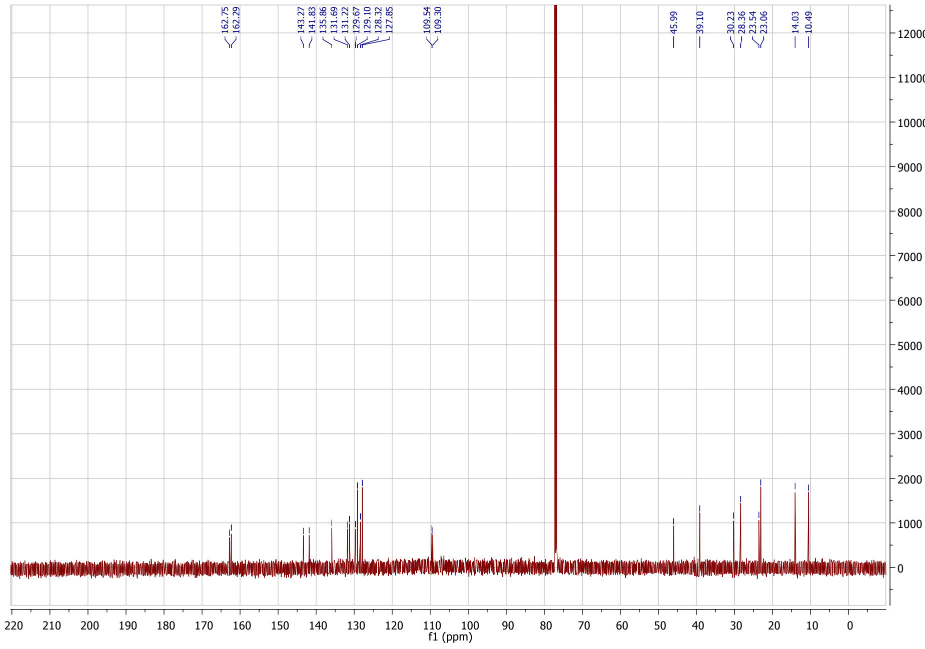

^{1}H NMR Spectra for **5bd**

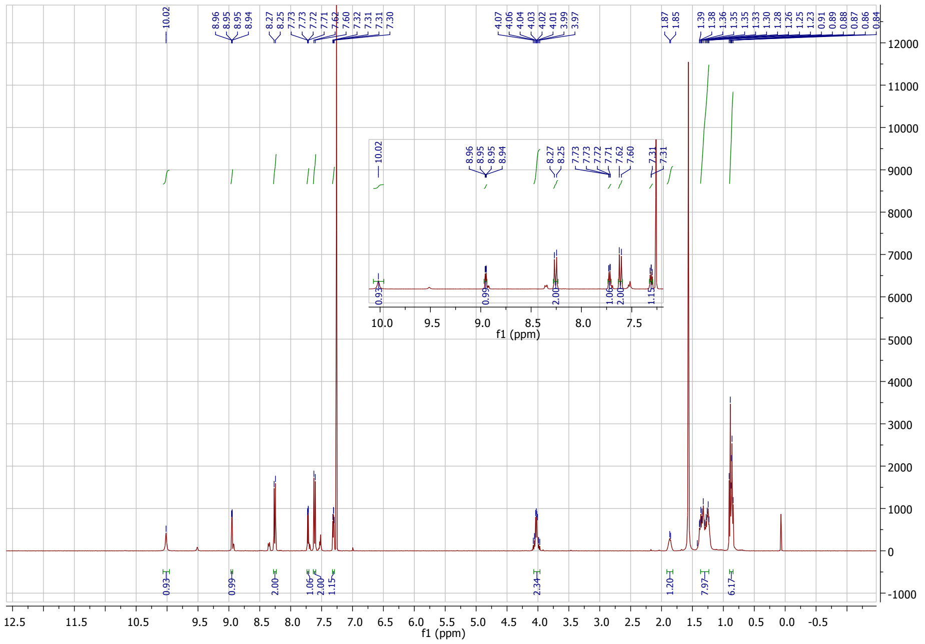

^{13}C NMR Spectra for **5bd**

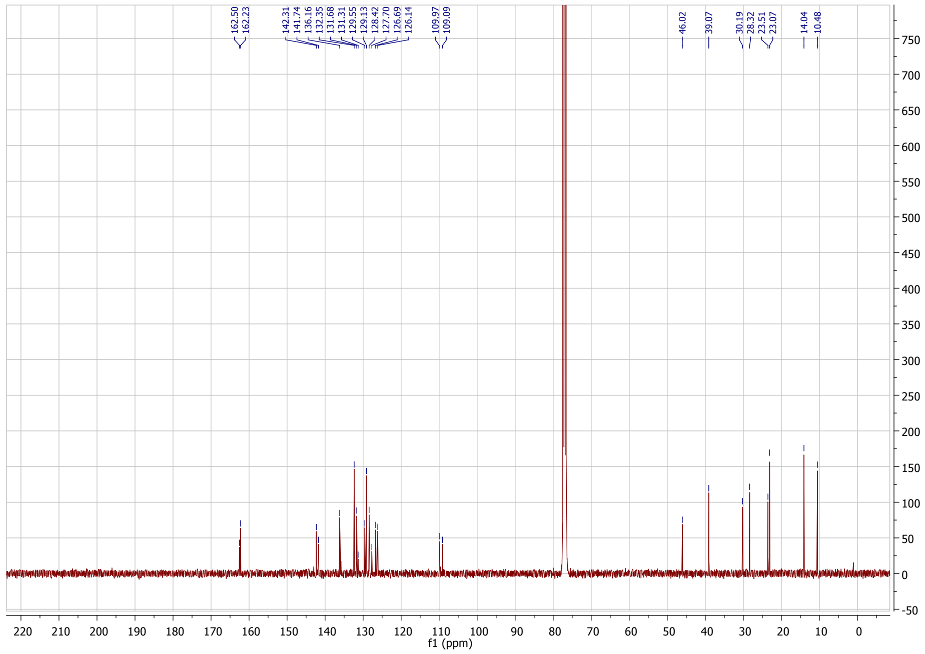

^{1}H NMR Spectra for **5be**

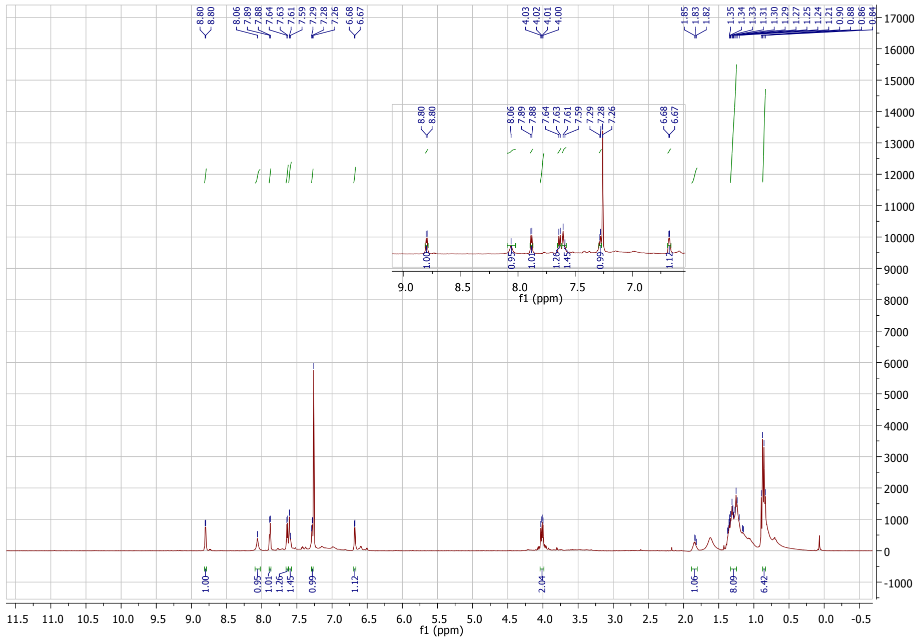

^{13}C NMR Spectra for **5be**

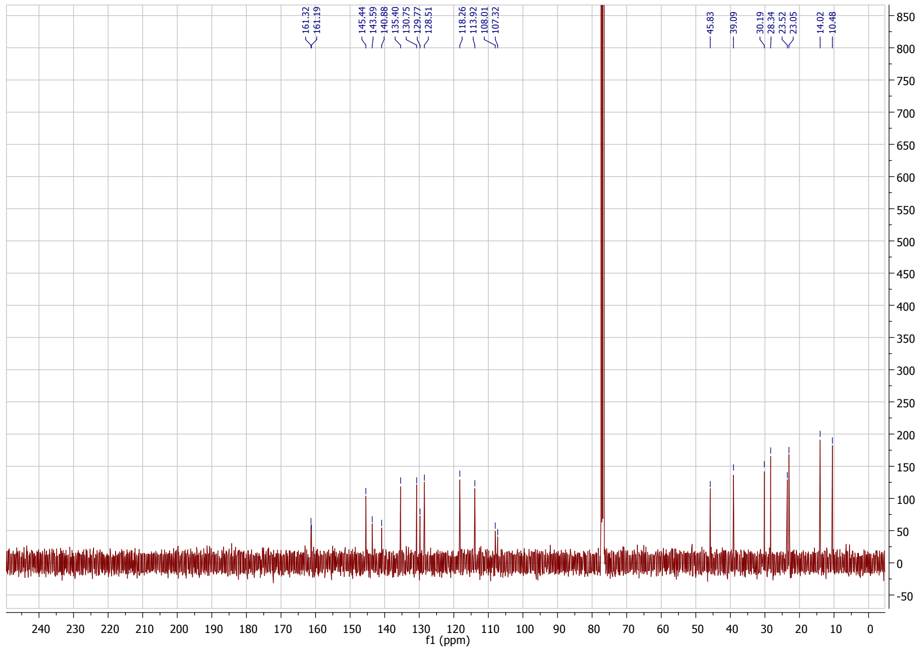

^{1}H NMR Spectra for **6adh**

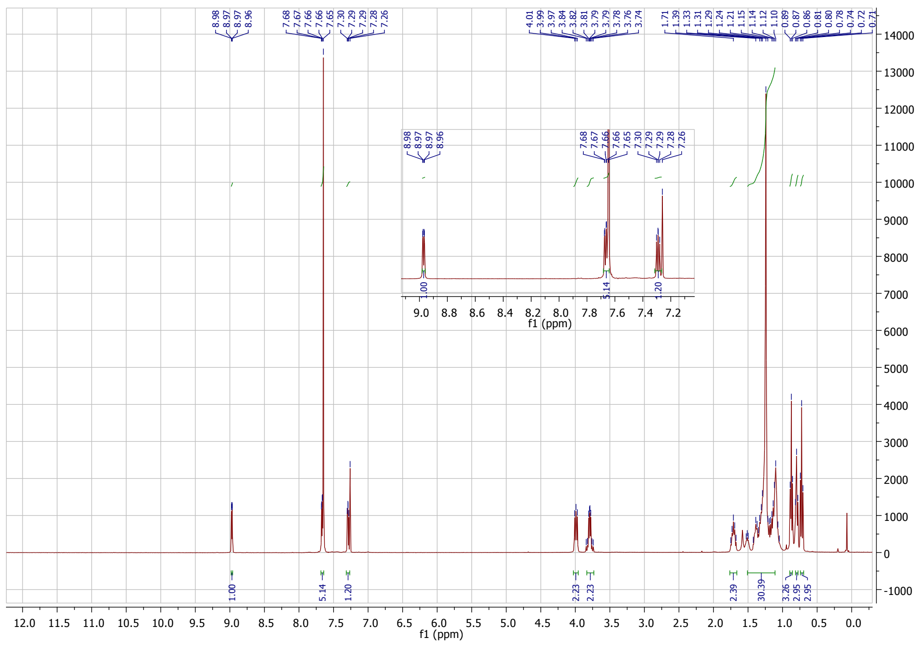

^{13}C NMR Spectra for **6adh**

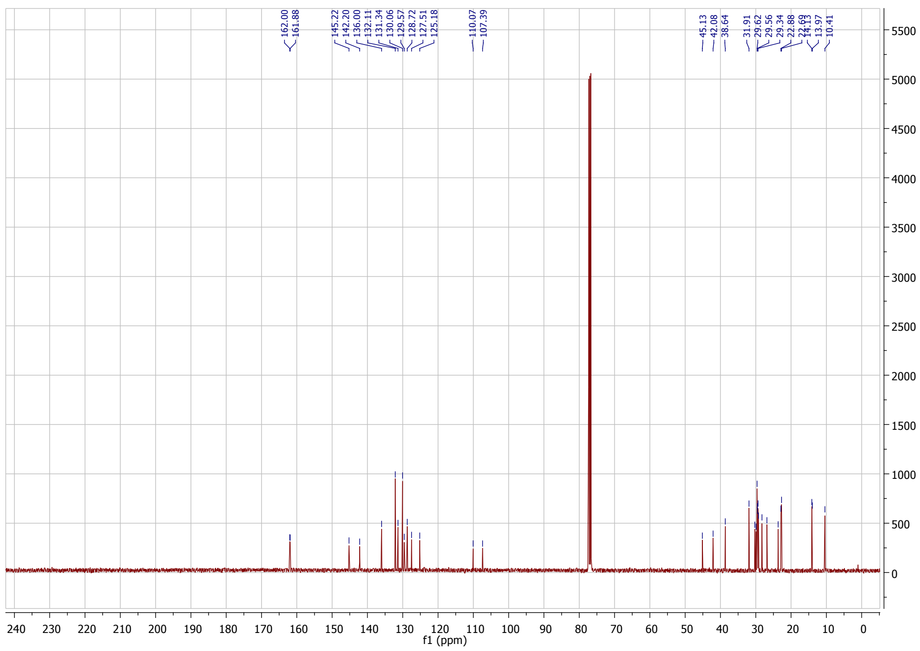

^{1}H NMR Spectra for **6adi**

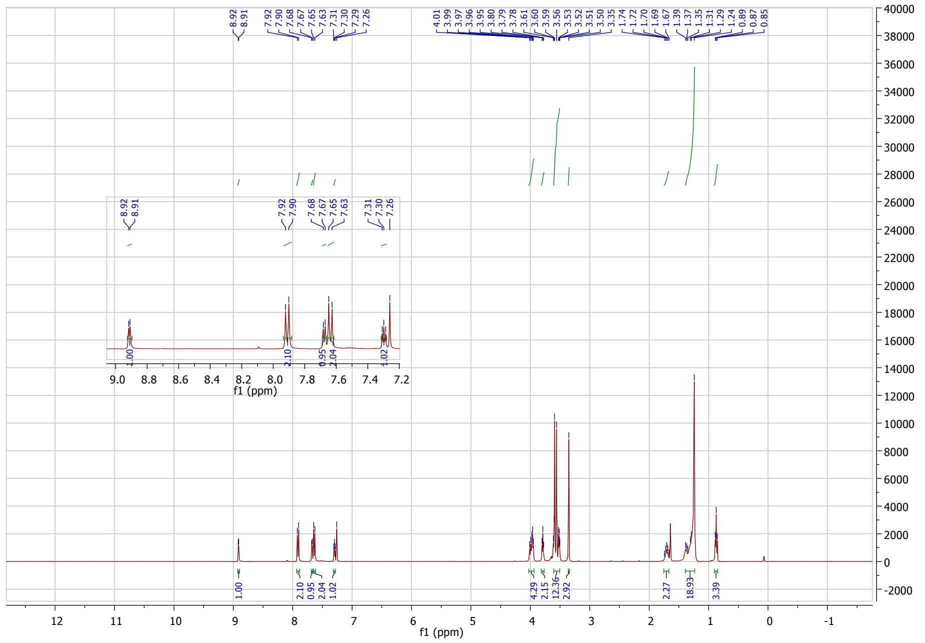

^{13}C NMR Spectra for **6adi**

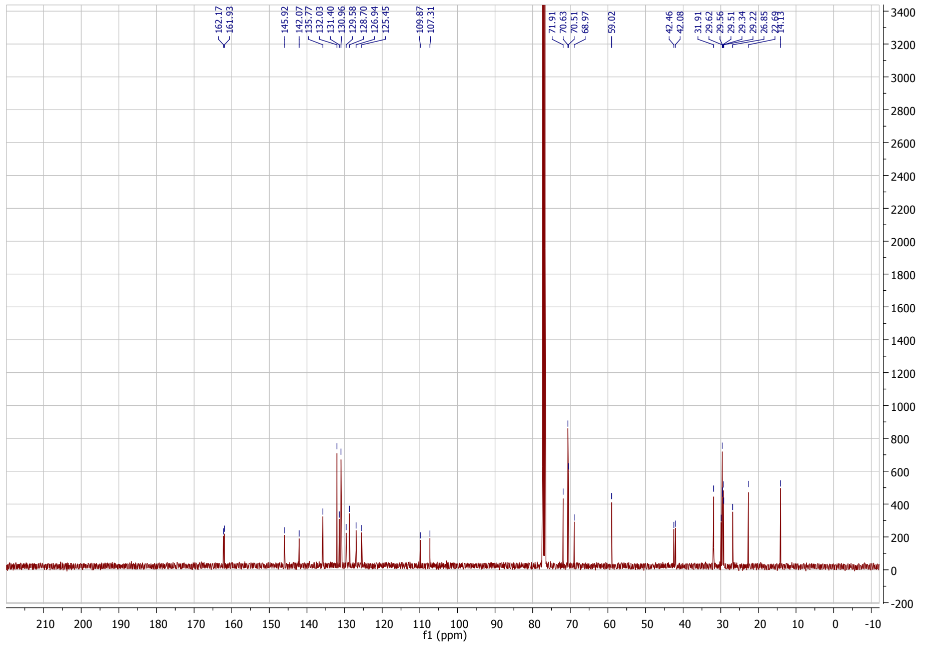

^{1}H NMR Spectra for **6bdf**

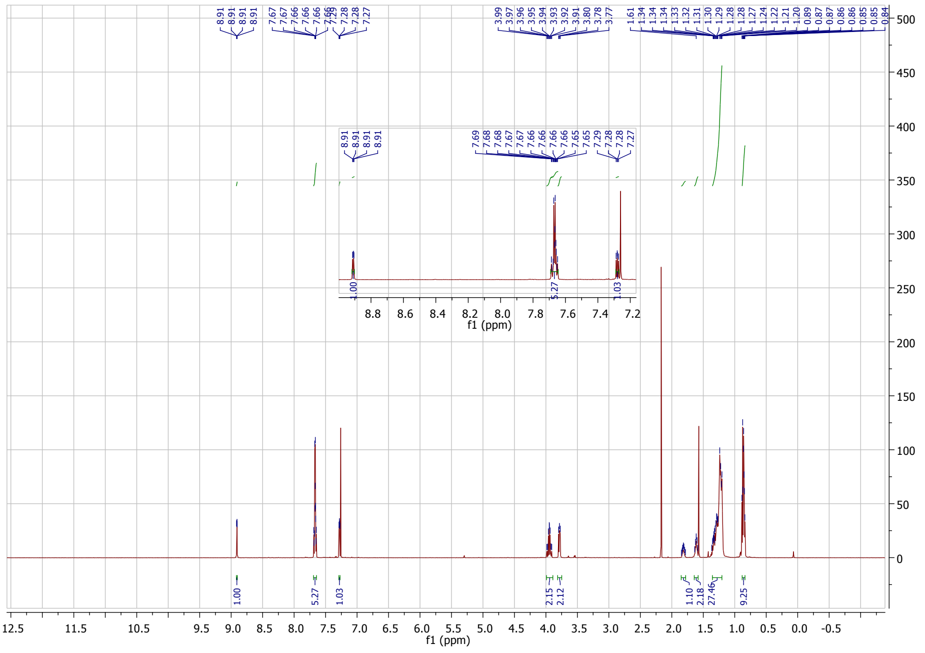

^{13}C NMR Spectra for **6bdf**

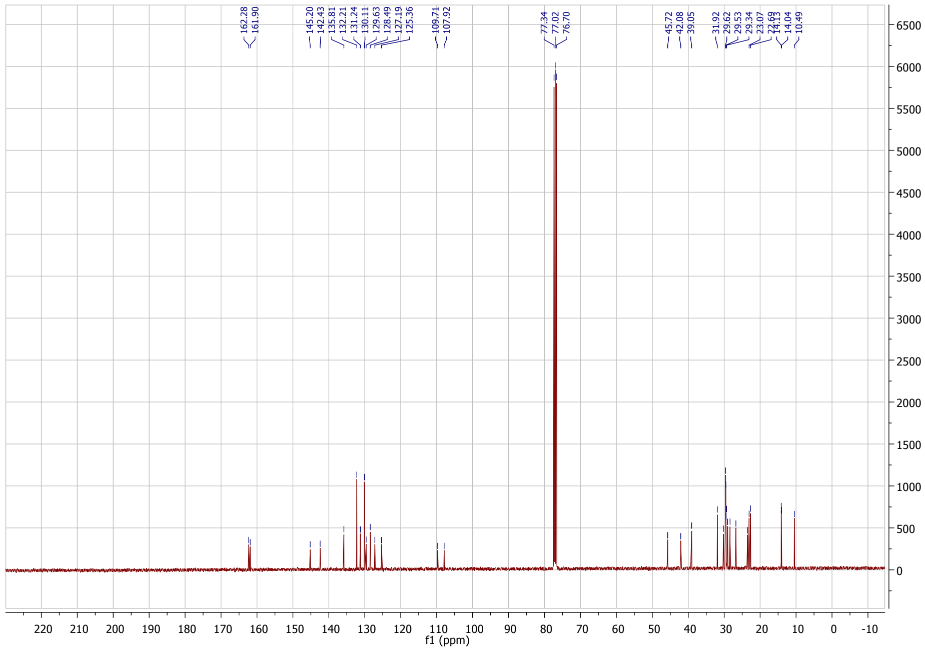

^{1}H NMR Spectra for **6beg**

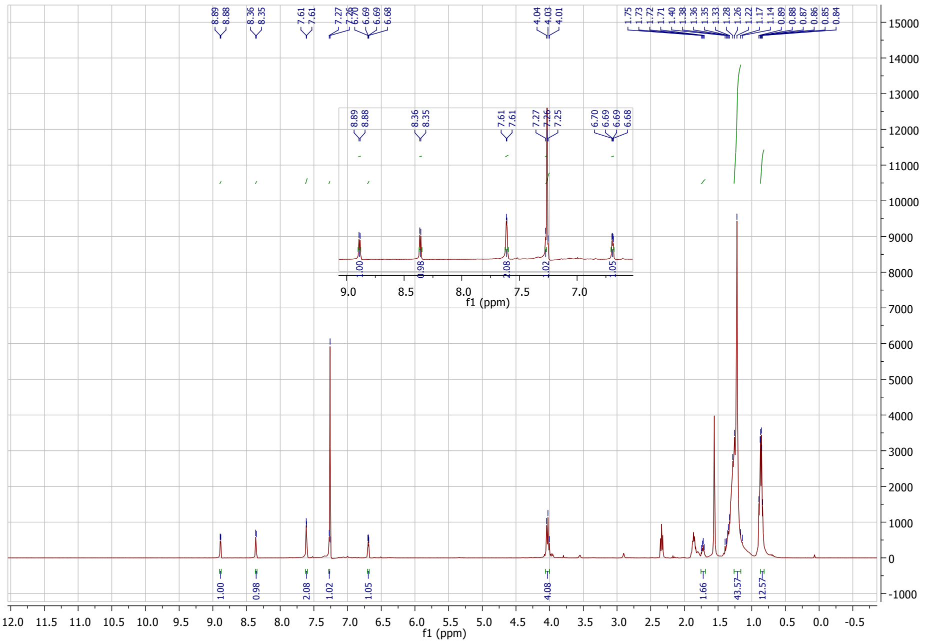

^{13}C NMR Spectra for **6beg**

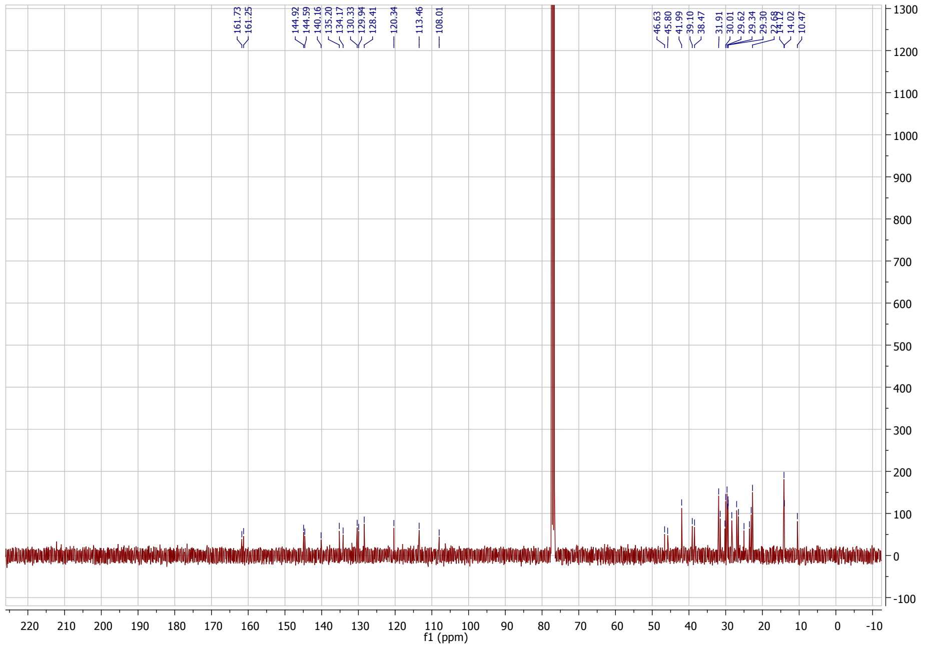

DFT Calculations

4. Geometry Optimised Structures, Molecular Orbitals and Coordinates

Ground state geometries are optimized at the B3LYP/def2-SVP level. [1] It is to be noted that the energy minimized structures have been obtained using a structurally modified model where both the alkyl chains have been replaced with methyl groups for simplicity.

HOMO

P-DPP

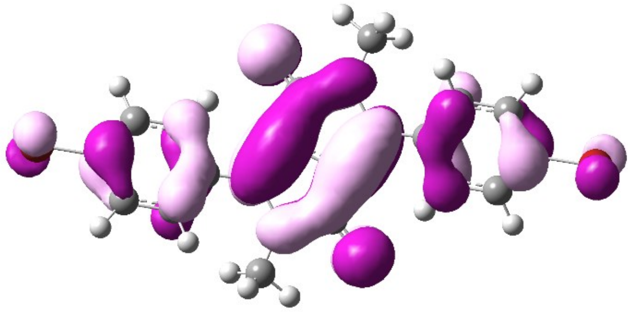

HOMO -5.51 eV

T-DPP-P/P-DPP-T

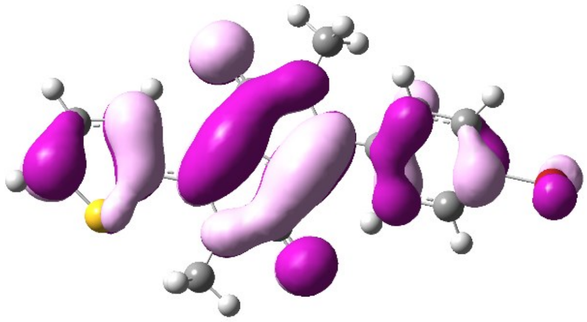

HOMO -5.32 eV

T-DPP

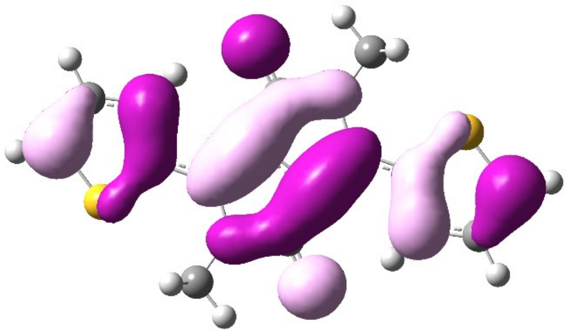

HOMO -5.13 eV

LUMO

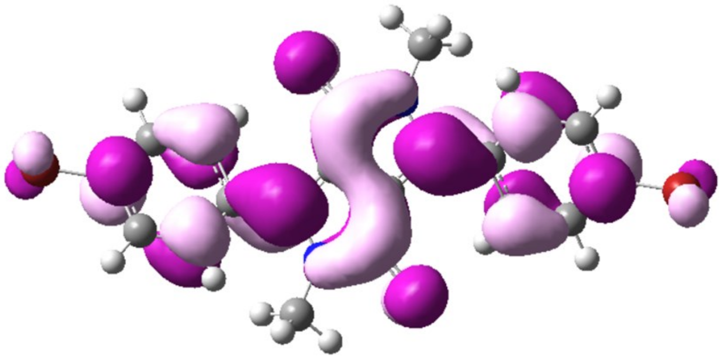

LUMO -2.86 eV

P-DPP

T-DPP-P/P-DPP-T

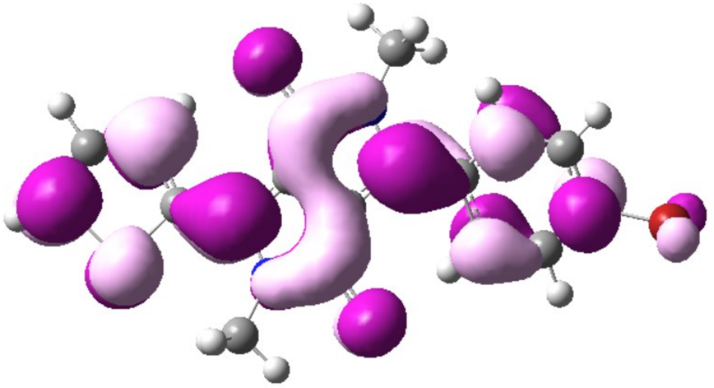

LUMO -2.78 eV

T-DPP

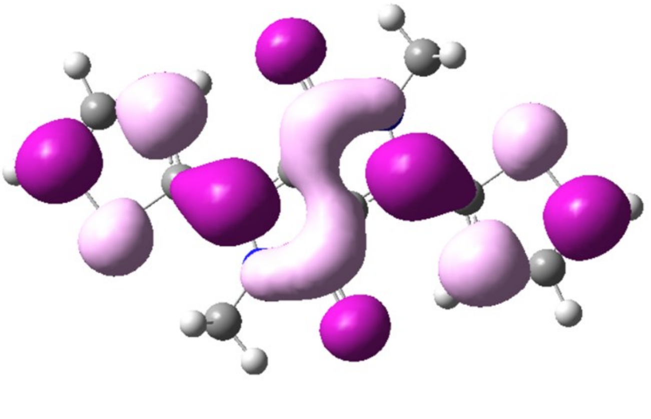

LUMO -2.68 eV

Coordinates

P-DPP

Charge = 0 Multiplicity = 1

O,0,3.3550051609,0.3043946703,10.5902430779

O,0,4.48107778,4.1244895366,14.9698253612

N,0,2.7570965849,0.4099744507,12.8423170778

N,0,5.2987301742,3.8578784703,12.8016828027

C,0,3.4515955225,0.8632895933,11.6699842619

C,0,2.9676679855,1.264593033,13.9235020741

C,0,3.7839392345,2.2990545971,13.4794478204

C,0,4.5027246472,3.4789460592,13.9352500812

C,0,5.0850751842,3.0055088064,11.7193281109

C,0,4.1365951631,2.0679290241,12.1128974819

C,0,1.8118761253,-0.6811775643,12.720529902

H,0,1.6262265998,-0.8214300895,11.6472674728

H,0,0.8693767408,-0.4443379652,13.2332234784

C,0,6.0155010803,5.1164046565,12.8362518678

H,0,5.6152827223,5.6859026404,13.6856786291

H,0,5.8644981014,5.6808757057,11.9057533015

H,0,7.0957212484,4.9780765552,13.0043559423

H,0,2.2122020006,-1.6277017283,13.1181438851

C,0,5.7775470466,3.09049083,10.4358987703

C,0,5.1895213646,2.4793693178,9.3058472997

C,0,7.0352054723,3.7095024676,10.2764199081

C,0,5.8156236754,2.5145793668,8.0640582899

H,0,4.2436846821,1.9469637932,9.4089905516

C,0,7.6704113407,3.7428075248,9.0373862367

H,0,7.550617933,4.1459344914,11.1285253955

C,0,7.052224561,3.1536126149,7.9315577941

H,0,5.3468214054,2.041450227,7.2004022108

H,0,8.6463369512,4.2181124622,8.9316980819

Br,0,7.9088735856,3.2095186736,6.2363520811

C,0,2.4415891459,1.0576712887,15.2704620551

C,0,2.0673510937,-0.2085643442,15.7673434218

C,0,2.3500072682,2.1667832931,16.1409500238

C,0,1.5956997891,-0.3616852977,17.0688415655

H,0,2.1713071858,-1.0987942087,15.1517953145

C,0,1.873739614,2.0218189011,17.4399617474

H,0,2.682143112,3.1488539783,15.8034245299

C,0,1.4902512502,0.7577104712,17.8982986293

H,0,1.3178652473,-1.3484556393,17.4411422796

H,0,1.8053699224,2.8885704717,18.098442035

Br,0,0.8336593379,0.555274095,19.6699032893

T-DDP-P/P-DPP-T

Charge = 0 Multiplicity = 1

S,0,2.7741708424,-0.1775841364,16.3303332706

O,0,4.703706294,0.0854435417,11.1239628604

O,0,1.7168957396,4.4464666487,13.7939433816

N,0,3.7573104762,0.3519403996,13.2388360885

N,0,2.7836289285,4.2396608382,11.7273197922

C,0,4.1088265131,0.7947208867,11.9174892686

C,0,3.0392474606,1.3217357469,13.929856215

C,0,2.9062094462,2.4207192906,13.0804022272

C,0,2.3792231244,3.7678897202,13.0218004522

C,0,3.5003972334,3.2691791793,11.0285209595

C,0,3.5605567455,2.1409065758,11.8424700403

C,0,2.5349404033,1.2066549477,15.2733310222

C,0,1.7906854225,2.1864665799,15.9251993409

H,0,1.5340976805,3.1313501691,15.4408024193

C,0,1.4267603637,1.8212085866,17.2442438987

H,0,0.8429594573,2.4570533685,17.9103914434

C,0,1.8876329484,0.5737940366,17.6001009318

H,0,1.752325113,0.0506891996,18.5456649343

C,0,4.1195241275,-0.9786038462,13.6734745808

H,0,4.6393305164,-1.4493363507,12.8283315134

H,0,3.2318397871,-1.5779482175,13.9281379293

C,0,2.2825675161,5.5147924442,11.2584315854

H,0,1.4611060528,5.7977593941,11.929995421

H,0,1.9101417113,5.4372364823,10.2275499202

H,0,3.0453962287,6.3086550593,11.3089247783

H,0,4.7977492763,-0.9526957426,14.5404563846

C,0,4.087343905,3.447620104,9.7025407404

C,0,4.4048767145,2.3000798596,8.9412558357

C,0,4.4021447298,4.7103375342,9.1571387347

C,0,4.9788444196,2.4107139612,7.6789975402

H,0,4.2239997049,1.308098222,9.3555421609

C,0,4.9822218055,4.8281980332,7.8961597901

H,0,4.2271244885,5.6200061667,9.7258571904

C,0,5.2586778865,3.6766566231,7.1554709403

H,0,5.2134592192,1.5143492095,7.1035433486

H,0,5.2259193063,5.8117608425,7.492655366

Br,0,6.0350158218,3.8336100515,5.4275813532

T-DPP

Charge = 0 Multiplicity = 1

S,0,3.8217313065,0.9393752616,16.9322025824

S,0,3.2330893953,4.2995735158,8.3753328153

O,0,6.0217136083,1.2510447934,11.8318932733

O,0,1.033419841,3.988404104,13.4757806599

N,0,4.8078534033,1.3642614956,13.8237916922

N,0,2.2472598103,3.8751550908,11.483863692

C,0,5.0546000448,1.6802276308,12.443858747

C,0,3.64706181,1.9728185192,14.2898218118

C,0,3.1093266042,2.6997550877,13.2258383623

C,0,2.0008442981,3.5596901174,12.8639831277

C,0,3.4084686047,3.2672332904,11.0180571824

C,0,3.9468718754,2.5413029845,12.082393281

C,0,3.1081756169,1.8675923812,15.6207319341

C,0,1.9408396519,2.4909643769,16.0536948975

H,0,1.3427341768,3.1183041211,15.3890620023

C,0,1.6372311213,2.2210177191,17.4107005862

H,0,0.7640264097,2.6204290208,17.9274333349

C,0,2.5623027538,1.3988622573,18.0129466511

H,0,2.5733663919,1.0352085933,19.0394072133

C,0,3.9469686243,3.3718088584,9.6869426769

C,0,5.1141780122,2.7483241044,9.2539265661

H,0,5.71250551,2.1213274328,9.918687444

C,0,5.4174584047,3.0177604324,7.8967431809

H,0,6.2906136035,2.6182590726,7.3799955951

C,0,4.4922695277,3.8397400404,7.2944550005

H,0,4.4809894792,4.2031051044,6.2678927364

C,0,5.7240857563,0.5074920102,14.5423294201

H,0,6.5129529681,0.2315526645,13.829796377

H,0,5.2284375769,-0.4081649962,14.9004734117

C,0,1.3315670705,4.7327280736,10.7655903549

H,0,0.542773486,5.008787299,11.478159655

H,0,0.8747835144,4.2130036105,9.9090966589

H,0,1.8277862665,5.6482489621,10.4078706382

H,0,6.1806452653,1.0278705601,15.3985512676

5. References

S1 S. Grimme, J. Antony, S. Ehrlich and H. Krieg, *J. Chem. Phys.*, 2010, **132**.
